# Abstracts of 33^rd^ Annual Conference of ISBTI: 33^rd^ ISBTI National Conference 5–7^th^ December 2008, SGPGI, Lucknow

**Published:** 2009-01

## FREE PAPERS

### Transfusion transmitted infections

#### To evaluate and quantify HCV RNS in low anti HCV antibody titer in blood donors and its possible transmission through blood transfusion – role of nat testing

**A. Chucklaira^1^, M. Dodwani, G. N. Gupta**

Santokhbai Charitable Blood Bank, Jaipur, India

The study of 44 sample showing low/borderline positivity on Enhanced chemiluminiscence (0.9 to 8) which were further subjected to HCV RNA quantification to study the possibility of transmitting HCV through this blood was done in our blood bank. The prevalence of anti HCV antibody among blood donor has been over estimated in recent year further higher rate of false positivity in Enzyme Immunoassay and Enhanced chemiluminiscence explain that only a minor part of EIA positive blood unit transmitted HCV to recipient. In our study done at SDMH, Blood Bank, Jaipur shows that only 18% of low positive anti HCV ECI results are HCV RNA positive, and 82% are false positive. In low positive anti HCV blood donors most results will be false positive. In such cases HCV RNA (which can identify false positive reaction) should be the test of choice. To avoid this waste and apprehension in blood donor should HCV RNA (NAT test) be the test of choice, can EIA result be overlooked in these cases.

#### Evaluation of sensitivity of three 4^th^ generation ELISA assays for detection of HIV i/ii and of 3^rd^ generation with 4^th^ generation elisa assays

**N. M. Bhatnagar, P. P. Thaker, A. S. Agrawal, M. R. Mittal, M. D. Gajjar, B. H. Parmar**

Department of Immunohematology and Blood Transfusion, Civil Hospital, Ahmedabad, India

*Background:* Serological evidence of HIV infection may be obtained by testing for HIV antigen or antibody in serum of individuals suspected of HIV infection. In order to reduce the window period and to increase assay sensitivity, new screening tests have been developed which simultaneously detect antigen (p24) and antibody. Although there are numerous studies carried out in the world over to show that these new assays are more sensitive in comparison to the former generation in reducing the diagnostic window by few days, the performance of these assays in individual set ups has to be evaluated.

*Materials and Methods:* A total of 717 donor samples were tested by both 3rd generation and 4th generation ELISA assays and the results were compared. Among these, 5 HIV reactive samples were serially diluted and tested by both ELISA assays.

*Results:* The results showed that the 4th generation ELISA assays could detect a lower level of antibody than 3rd generation assays. Also, three different 4th generations ELISA assays were compared for their performance which showed equivocal performance.

*Conclusion:* Thus, for the detection of both established and early HIV infection, use of 4th generation ELISA assays as a screening test should be made mandatory in all blood banks to ensure complete blood safety.

#### Comparison of particle gel immunoassay (PAGIA) with t.Pallidium particle agglutination (tp-pa) test and VDRL in donor and patient sample for serodiagnosis of syphilis

**A. Joshi, K. Saluja, B. Thakral, S. Sethi^1^, M. Biswal^1^, A. Jain, R. Sharma, N. Marwan**

Departments of Transfusion Medical and *Medical Microbiology PGIMER, Chandigarh, India

*Background:* Serological test for treponemal antibodies are essential for the diagnosis of syphilis in donors as well as patients. Non treponemal tests such as VDRL and RPR though sensitive in early syphilis, have disadvantage of false positive reactions, false negative reactions due to prozone phenomenon and lack of sensitivity in late stage infections. Treponemal tests such as MHA-TP and TP-PA show high sensitivity for all stages of disease other than very early primary syphilis. PalAs have been introduced for detection of infectious disease in addition to red cell serology. It consist of a microtube containing a gel matrix and red polymer particle sensitized with recombinant antigens TpN15, TpN17 and TpN47 in a ready to use suspension. We compared the result obtained from PAGIA with VDRL and TP-PA.

*Materials and Methods:*70 samples including 50 donors and 20 patients which were reactive with VDRL were tested again with Serodia TP-PA test and Diamed PaGIA. Briefly 10 microlitre of serum or plasma was pippetted into the funnel of appropriate PaGIA microtube and 50 microlitre of vortexed particles was added. Mixture was incubated at room temperature for 10 minutes and results read visually.

*Results:* Out of 50 donor samples reactive with VDRL 86% (n=43) were also positive with PaGIA, 10% (n =5) were negative and 4% (n=2) were indeterminate. 84%(n=42) were positive with TP-PA, 14% (n=7) were negative while 1 was indeterminate. Out of 20 patient samples reactive with VDRL 95% (n=19) were also positive with PaGIA and 5% (n=l) was negative. For donor samples PaGIA had a sensitivity of 100%, specificity of 83.3% and a total predictive value was 95%. Correlation with TP-PA was 0.688. Overall sensitivity and specificity considering 70 samples was 100% and 75% and total predictive value was 97.05%. Correlation with TP-PA was 0.852.

*Conclusion:* PaGIA shows an excellent sensitivity compared to TP-PA. IN comparison to TP-PA, a reaction time of only 20 minutes is needed and loading of samples is easier. Thus it can be a good choice for screening of donors and patients of detection of T.Pallidum

#### Use of NAT and an enhanced chemiluminescence technology in screening blood donors for HCV, HIV and HBSAG

**R. Menon, K. Marimuthu, R. Selvi Priyadharshini**

*Background:* This study is undertaken to explore the utility of using two sensitive methods in screening blood donors for the Transfusion Transmitted Disease (TTD) markers in a blood bank in order to ensure blood safety.

*Materials and Methods:* Nucleic acid testing was done using Chiron's Procleix Ultrio Assay and Discriminatory assay kits for HCV, HBV and HIV; VITROS enhanced Chemiluminescence method was used for screening for aHCV, aHIV and HBsAg. A total of 4500 donors attending the blood bank at Apollo Hospital were screened by both methods.

*Results:* From the results that have been obtained, there were discrepant results on three samples; one of three was positive by NAT but negative by ECi methods; the other two discrepant results were negative by NAT but positive by enhanced chemiluminescence method; one of the two was positive for VITROS aHCV and confirmed by RIBA HCV; the other was confirmed positive for VITROS HBsAg. No discrepancies noted for HIV so far, between the two methods.

*Conclusions:* It is obvious that neither of the sensitive methods can be used in isolation for ensuring blood safety. Use of NAT along with a sensitive method for aHCV, HBsAg and aHIV can be useful in detecting the viruses/antibodies, thereby ensuring blood safety.

#### Prevalence, trend and co-infection of infectious disease markers in blood donors

**N. S. Ingole, A. Thakre, D. Joshi, N. Gangane**

Department of Pathology, Mahatma Gandhi Instituite of Medical Sciences Sevagram, India

*Background:* Transfusion transmitted diseases (TTD) are a major challenge to transfusion services all over the world. This study was undertaken to find out the prevalence, trend and correlation between HIV, HBsAg, HCV and VDRL positivity in the blood donor population in Wardha district.

*Materials and Methods:* Data for the present study was obtained from blood bank registry of TTD positive donors at MGIMS, Sevagram who were selected for blood donation between the period of 1st January 2001 to June,2007. The screening for anti HIV I and II, HBsAg, and anti- HCV was done by ELISA whereas VDRL testing was done by Rapid Plasma Reagin test marketed by Tulip diagnostics.

*Results:* The percent prevalence rate of HIV, HBsAg, HCV and VDRL reactivity was found to be 1.1,2.3,0.87 and 0.84 respectively amongst total 26603 donors bled during this period. The statistical analysis of co-occurrence of TTD markers showed highly significant positive correlation of HIV with VDRL but not with HBsAg and HCV. There was significant decrease in the prevalence of HbsAg from 2001 to 2005, but it again increased in the last two years because of more reactivity of HBsAg in replacement donors.

*Conclusion:* In spite of rigid donor selection criteria and screening for TTD markers, there is some risk of post transfusion infection, hence transfusion of blood or blood products should be done when it is utmost indicated.

#### Individual donor NAT- an established global standard for blood safety

**R. N. Makroo**

Transfusion Medicine, Indraprastha Apollo Hospitals, New Delhi, India

*Background:* As transfusion transmitted infections (TTI) can be transmited through infected blood, considerable efforts has been devoted in recent years to reduce their transfusion transmission worldwide. Indraprastha Apollo Hospitals evaluated NAT in 2005 with more than 12,000 samples and started routine NAT screening from April, 2006. With the success of this technology towards TTI free blood supply, other Apollo Group Hospitals like Apollo Hospitals Chennai including Apollo Cancer Hospital and Apollo Hospitals Bangalore Hospitals implemented NAT.

*Materials and Methods:* NAT evaluation and routine NAT is being performed using Chiron's Procleix Ultrio Assay on Procleix eSAS system. Procleix Ultrio Assay is the first multiplex, single tube, highly sensitive NAT screening method for HIV-1, HCV and HBV.

*Result:* NAT Evaluation: Between June 2004 and January 2005, 12,224 unlinked samples were collected from 8 different blood banks. Among 12224 samples there were 8 NAT yield cases: 1 HIV, 1 HIV-HCV co-infection, and 6 HBV. Our observed NAT yield for all 3 viruses is 1 in 1528. Routine NAT Indraprastha Apollo Hospitals New Delhi: Routine NAT was implemented in April 2006. Among 49,419 samples tested till October 2008, 13 NAT yield cases: 5 HBV, 1 HCV, 3 HBV (HBsAg Neg but HBcAb Pos), 1 HBV- HCV co-infection (Only HBsAg Pos), 1 HIV-1 - HBV Co infection (HIV Ab and HBcAb Pos) and 2 HCV (HBcAb Pos). Our observed NAT Yield for all the three viruses is 1 in 3801. Routine NAT in Apollo Hospitals Chennai: Apollo Chennai implemented routine NAT in January 2008. It is a centralized NAT screening facility also used by Apollo Cancer Hospital and Apollo Hospital Bangalore. Among 11,154 samples tested till October 2008 5 HBV NAT yields were found. Our observed NAT yield rate for all the three viruses is 1 in 2231.

*Conclusion:* NAT has increase the blood safety of Apollo group of hospitals. Since its implementation there are no reported cases of Transfusion Transmitted Infections. This results in the increased confidence in our blood supply among general public, blood bank staff. A total of 18 potential TTIs detected and intercepted in two years or prevention of approximately 30 potential cases of infection through different blood components. Experience of other countries like South Africa confirms that Individual Donor NAT can prevent TTIs and make blood safer.

**Table 1:** NAT Yield data

**NAT**	**Samples Tested**	**Yield**	**Yield rate**
NAT evaluation Study	12224	8	1 in 1528
Indraprastha Apollo	49419	13	1 in 3801
Hospitals (Routine)Apollo Hospitals	11154	5	1 in 2231
Chennai (Routine)Total	72797	26	1 in 2800

#### Trend of HIV positivity in the blood bank of a tertiary referral hospital in South India

**N. Reagan, M. Rajaiah, Purna M. S. C. Nair, D. Daniel**

Department of Transfusion Medicine and Immunohaematology Christian Medical College, Vellore, India

*Background:* The reported prevalence of HIV positivity in the Indian donor population has ranged from 0.08% to 3.87%. Enhanced quality of available kits and more stringent donor screening help to decrease infectious disease positivity. We report on the trend of HIV positivity among blood donors over a 10 year period in a tertiary referral hospital in South India.

*Aim:* To study the trend of HIV positivity among blood donors over a 10 year period (April 1998 to March 2008). To see if increasing numbers of voluntary donations and introduction of pre donation counseling had an effect on the trend of HIV positivity among eligible donors.

*Materials and Methods:* The HIV screening results of all consecutive donors from 1998 to 2008 were reviewed including the confirmatory Western Blot. Correlation between increasing voluntary donors and the trend of HIV positivity was looked at and so also the effect of stringent donor screening.

*Results:* The study reveals decreasing trend in HIV positivity among donors, from 4.4 per1000 in 1998-1999 to 1.7 per 1000 in 2007-2008. The percentage of voluntary donors showed a significant increase from 11% in 1998 to a maximum of 26% in 2007. The increase has contributed to this trend of decreasing HIV positivity so also has stringent pre donation counseling reinforced in 2003-2004. The overall ELISA positive were 769 (0.43%) and Western blot positive were 513 (0.29%) of which 509 (99.22%) were HIV Type I and 4(0.78%) were HIV Type II respectively.

*Conclusion:* Though there is an overall decrease in HIV positivity overtime, the prevalence of HIV among blood donors remains a cause for concern. Despite more sensitive methods for HIV detection, we must rely on stringent screening of donors through accurate history taking to decrease the risk of transfusion transmitted HIV.

#### HIV seroconversion in multi-transfused thalassemic children

**S. Naseem, R. R. Sharma, N. Marwaha, A. Trehan, R. K. Marwaha**

Post Graduate Institute of Medical Education and Research, Chandigarh, India

*Background:* Transfusion-transmitted infections (TTI) among multitransfused patients such as thalassemia continue to be a major challenge for blood transfusion organizations across the world. Although with the advent of donor screening practices, reported incidence of TTI have decreased, but they can still get transmitted because of inappropriate donor selection or during the window period. This study was carried out with the aim to determine the prevalence of seropositivity of HIV among Thalassemic patients receiving regular transfusions

*Materials and Methods:* The case records of patients with Thalassemia enrolled in the 'Thalassemia Transfusion Programme' at Advanced Paediatric Center, PGIMER, were retrieved and analyzed.

*Results:* Under the 'Thalassemia Transfusion Programme' which started in 1986, a total number of 339 Thalassemic patients (239 males, 90 females) have been enrolled till date. These patients receive transfusion on a regular basis at intervals of 15-28 days and around 6,000 packed red blood cells are transfused to them in a year. All these patients had received first transfusion between ages of 3-12 months. Regular mandatory screening for TTI - HIV, HBV and HCV is carried out in our institute and screening for HIV commenced in 1994. Over the years only 3 (0.8%) children have tested positive for HIV. Of these 2 are males and 1 female, at present aged 9, 14 and 18 years old and are receiving apart from regular transfusion, anti-retroviral therapy and have no manifestation of HIV disease.

*Conclusion:* The prevalence of HIV infection was found to be low; seroconversion was seen in 0.8 % patients. Rarity of seroconversion revealed by our results indicates that the currently used practices for donor selection and plasma products used are safe. However, in spite of an established HIV screening programme 4 patients became HIV positive, which may be attributed to transfusion of blood from donors during the window period (90 days by ELISA). With the newer direct tests of viral gene amplification [nucleic acid amplification testing (NAT)], this window period gets truncated (9 days); these tests have become a routine part of blood donor infectious screening in developed countries over the last decade.

#### Screening donated blood for malaria parasite using PLDH based rapid malaria test

**P. Shrivastava, S. Kumar, S. P. Tanwar, S. Sarkania**

ESI Hospital, Basai DaraPur, New Delhi, India

*Background:* Malaria is caused by the bite of infected female anophelese mosquito. It can also be transmitted through blood transfusion. Testing for malaria parasite in donated blood is mandatory as per drugs and cosmetic act part XII B of schedule F. Blood bankers face a lot of difficulties in fulfilling this criteria. We present our experience of using pLDH based rapid malaria test for the purpose.

*Materials and Methods:* All the donated blood units were screened for malaria parasite using OptiMal Rapid Test. Thick and thin smears of all positive cases were examined microscopically to co-relate the results of rapid test. Blood units found positive for Malaria were discarded and donors were notified of the result.

*Result:* Total 15125 units were screened by OptiMal rapid test. Three units (0.02%) were found to be positive; two for *P. vivax* and one for *P. falciparum.* Both the cases of *P. vivax* were positive on microscopy but the one with *P. falciparum* was negative. None of the donors had given history of fever or malaria during pre-donation interview. All the three donors were notified. Only one donor (Pf +) came back. A fresh blood sample was drawn which was positive for *P. falciparum* by OptiMal test. Microscopic examination of many slides revealed only one trophojoite form in one of the thin smears. He was referred to medical OPD for anti-malarial treatment.

*Conclusion:* Transfusion associated malaria should be considered in differential diagnosis of a febrile illness following blood transfusion in any patient. Although incidence of malaria parasite in blood donors in our study is very low, it reflects that steps must be taken to prevent infectious units from entering the blood supply by improving testing techniques and donor awareness. There is need for adopting accurate, sensitive and cost-effective rapid tests for screening of malaria in Blood Banks.

#### Prevalence of HIV I/II, HCV, HBSAG co – infection in blood donors

**A. S. Agra wal, N. M. Bhatnagar, M. R. Mittal, J. T. Jambukiya, M. D. Gajjar, B. H. Parmar**

Department of Immunohematology and Blood Transfusion, Civil Hospital, Ahmedabad, India

*Background:* Infection by the hepatitis B (HBV) and hepatitis C (HCV) viruses is the most common cause of post transfusion hepatitis. However, with the emergence of HIV infection more emphasis has been given to the control of blood utilized in transfusions. Studies show that a co-infection by HBV and HCV is as frequent in Asia, as it is in western countries, varying from 10% to 15% in patients who are chronically infected by HBV. The high prevalence of HBV and HCV may result in an increase in the risk of transmission of these viruses through the transfusion of hemocomponents, since it is not possible to totally guarantee the absence of these infections among blood donors through the serologic tests utilized routinely in the screening of blood donors. We evaluated the prevalence of HBsAg, anti-HIV-I/II and anti-HCV co-infection in voluntary and replacement blood donors.

*Aims:* To investigate the prevalence of HIV-I/II, HCV, HBsAg co-infection in voluntary and replacement blood donors.

*Materials and Methods:* A prospective and retrospective study was done in the Department of IHBT, BJMC and Civil hospital Ahmedabad during 1996- 2008. Donors were selected according to standard criteria recommended by National Aids Control Organization.

*Results:* A total of 1, 80,000 blood donors were included in the study. Among these donors, 782 were HIV-I/II positive, 357 donors were HCV positive, and 1793 donors were HBsAg positive. All donor samples were tested by ELISA. Number of donors having co infection was 18.

*Conclusion:* The present study emphasizes the continuing need for an effective donor screening program to protect blood recipients and keep transfusion transmitted diseases to minimum. Presence of co-infection in blood donor can be fatal if transfused to recipient in cases of non-detection in window period. Recruitment and retention of Voluntary donors is the key to a safe blood supply

#### Prevalence of cytomegalovirus reactivity in blood donors in a tertiary care hospital

**N. M. Bhatnagar, A. Shah, A. S. Agrawal, M. R. Mittal, M. D. Gajjar, B. H. Parmar**

Department of Immunohematology and Blood Transfusion, Civil Hospital, Ahmedabad, India

*Background:* Cytomegalovirus has emerged as a significant cause of morbidity and mortality following blood transfusion in children and immunocompromised adults. In India, it is not mandatory to screen blood donors forCMV. Very few studies have been conducted in India regarding the seroprevalenceofCMV among donors. The current study was undertaken in an attempt to address this aspect.

*Materials and Methods:* The study population consisted of 223 donors, both voluntary and replacement. Blood samples of all 223 donors were collected, sera separated and tested for both CMV (IgG) and CMV (IgM) by ELISA method.

*Results:* Out of 223 donors, 171 (76.68%) were voluntary and 52 (23.32%) were replacement donors. There was no statistical difference in CMV reactivity in the two groups. The percentage seropositivity of CMV (IgG) was 99.5% and CMV (IgM) was none.

*Conclusion:* CMV infection is highly prevalent in our country so, blood and blood products for neonates and immunocompromised patients should be screened for antibodies so as to prevent iatrogenic transmission.

#### Prevalence of hepatitis B and C in hemophilia patients of gujarat

**M. Mittal, N. Bhatnagar, M. D. Gajjar, B. H. Parmar**

Department of Blood Transfusion and Immunohematology, Civil Hospital, Ahmedabad, India

*Background:* Hemophilia is a genetic disorder in which there is deficiency of different coagulation factors due to which bleeding tendency is present in the patients. The most common type is hemophilia A in which there is deficiency of Factor VIII. Patients have to take the deficient factor by means of FFP or Cryo or factor concentrate. In India most of the hemophiliacs can not afford factor concentrate. FFP and cryoprecipitate are the main treatment for these patients.

*Materials and Methods:* For the study, 72 hemophilia patients were selected, of age group 1 year to 62 years. Blood samples of the patients were collected and were tested by ELISA for hepatitis B and C viruses and the reactive ones were confirmed by the repeat testing.

*Results:* Out of the 72 patients 6 patients (8.33%) were found reactive for HCV and 2 patients (2.78%) were reactive for HBsAg (one patient was reactive for both HBsAg and HCV). The reactive patients belonged to the age group 30-60 year. The total prevalence of both HCV and HBsAg was found to be 11.11% which is found to be high.

*Conclusion:* As these patients are multitransfused, so measures to identify donors in window period of these disease and proper donor selection is vital.

#### Serosurviellance of HBSAG in voluntary blood donors, sero markers, viral load of HBV and genotyping of the strain affecting these donors

**S. Pushkala**

Department of Immunology, TN MGR Medical University, Chennai, India

*Aims:* To find out percentage positivity of the HBsAg among voluntary blood donors and other sero markers in these donors. To estimate viral load in sero positive donors and genotyping of the strain affecting these donors.

*Materials and Methods:* ELISA screening of all donated blood. Positive samples subjected to other sero markers like HBeAg and HBcAb. Molecular method performed to estimate the quantity of the virus in the sample using Amplicor semi quantitative kit by thermal cycler. *Results:* HBsAg positivity in blood donors was 2.6% among 3000 voluntary donors. 1.2% had HBeAg positivity. HBcAb is yet to be done. Viral load in 73 % of positive patients is less than 1000 copies per ml.27% has viral load ranging from 2000 - 6000 copies per ml. Genotyping has yet to be carried out.

*Conclusion:* The patient who has other seromarkers positive should be strictly followed and should be under the Gastro enterologist 6 months sampling have to be carried out to see the progress of the patient who has 1000 or less copies / ml. The patient with > 1000 copies has to be treated with antiviral. Short study was undertaken to see that the semi quantitiave test helped profusely in determining the prognostic value of the anti viral treatment given to the patients. No correlations as yet could be drawn with the seropositivity and the viral load. Genotyping can be done to see there is a difference between frank disease patients and these asymptomatic patients.

#### Adapting to new horizons in TTI screening

**A. Shanker**

Artemis Health Institute, Gurgaon, India

*Background:* Blood Transfusion Services are challenged by the Transfusion Transmitted Infection esp. HIV, HBV and HCV. Infection screening has been done by Chemiluminescence (CLA; Antibody to HIV-1 and2, HBsAg, Anti- HCV and Antibody to Hepatitis B Core) in routine practice in addition to individual donor nucleic acid test ID- NAT (HIV-1, HBV and HCV)

*Materials and Methods:* This is a pilot study of ID-NAT and CLA for various parameters including changes in results with the experience of the person performing test, parameters of errors observation, inventory holding timing, trend analysis of the regent and performing of the test and LJ chart.

*Results:* 1011 samples have been tested. CLA has been positive for 1.48% without HB Core Antibody and 7.02% with HB Core Antibody. ID-NAT is reactive for 15 samples (1.48%). During the routine use, we have not observed any CLA negative and NAT positive. Confirmatory tests have shown same samples to be reactive in CIA as well as NAT. Borderline CIA were ID-NAT negative. The samples have been invalid 19 times, which has to be repeated. There has been a decline in the invalid rates over a time when technician has been trained. Evening shift had highest invalid tests (2.47%). Average inventory holding time has increased from 6 hours to 16 hours.

*Conclusions:* There are changes in the logistics that are necessary to bring with the implementation of ID-NAT. these include sufficient regent handling, continuous LJ plotting and repeat of the samples (as and when required) before the blood is released for the transfusion. Psychological benefit of use of ID-NAT in addition to CLA is not observed any ID-NAT positive which is CIA negative. This requires further evaluation of HB Core along with ID-NAT, needs to be reevaluated. Availability of EQUAS will be helpful in further improving the results.

#### Prevalence of seropositivity for HIV, HBV HVC VDRL and Malaria in south indian blood donors: A hospital based study from 2000 – 2008

**S. Bonagiri, R. G. Sastry**

Department of transfusion medicine Nizam's Institute of Medical Sciences, Hyderabad, India

*Background:* There is definite correlation between the Blood Donors and general population incidence of viral hepatitis and retroviral disease. The changing trends, with awareness among the people, vaccination, and new antibiotic regime are also seen in the incidence of Transfusion Transmitted diseases. It is very important to encourage the voluntary donors to cut down the professional donors.

*Materials and Methods:* In this study we have included the voluntary and Replacement donors, total 1,29,421 from January 2000 – June 2008. The donors were screened with fourth generation ELISA for HIV seropositivity and third generation ELISA for HBV and HCV infections. VDRL with RPR Method and Malaria with QBC and ELISA Methods.

*Results:* The Seroprevenlance of HIV was 0.5-1.01, HBS Ag 2.5-4.03, HCV-0.35-1.25, VDRL – 0.28-1.2HBC Ig- 16.23 – 23.08. AntiHBS Ag 5.5 -8.2 The incidence of HIV HBS Ag HCV Anti HBS Ag are decreasing. The seroprevelance of VDRL and HBC IG are on increasing side. All the Voluntary donors who were contributed for 5% of total donors are seronegative.

*Conclusion:* The seroprevalance of retroviral and HBV infections is decreased in these eight years. Prevalence of HCV is slightly decreased. VDRL Core Antibodies is increasing awareness among the public, vaccination and new antibiotic therapies. There is definite need to encourage voluntary donors to avoid transfusion transmitted infection.

#### Non-infectious complications of transfusion

**R. Krishnamoorthy, V. K. Panicker, R. S. Febe**

Sri Ramachandra University, Chennai, India

*Background:* Blood Transfusion is a life-saving measure; but it has inherent risks as well. Informed consent has to be obtained in writing from the patient or the guardian before ordering a transfusion. No blood is 100% safe in spite of stringent blood safety measures. The benefits of transfusion should outweigh the risks involved.

*Aim:* To study the frequency of acute non-infectious Transfusion Reactions in a tertiary level of care hospital.

*Materials and Methods:* The study was carried out at the Sri Ramachandra Medical Centre Blood Bank, Sri Ramachandra University, Porur, Chennai. Acute non-infectious Transfusion reactions that were reported to the blood bank following transfusion of blood components between April 2006 and July 2008 were analyzed.

*Results:* Incidence of acute non-infectious Transfusion reactions was 0.26% (88 out of 32929 Transfusions). Febrile non-hemolytic transfusion reaction (FNHTR) was the most common transfusion reaction encountered (50% of all Transfusion reactions) followed by allergic reactions (28.4%).Breathlessness was reported as a transfusion reaction (5.7%), chest pain and restlessness together contributed to 2.28% of reactions; Acute haemolytic transfusion reaction (AHTR) due to non-immune hemolysis occurred in 2.27% followed by nausea and giddiness in 1.14%. Packed Red Blood cells were the commonest blood component implicated (93.18%) in the causation of FNHTR. Platelet transfusions accounted for 4.55% and FFP accounted for 2.27% of FNHTRs. Acute transfusion reactions due to Fresh frozen plasma (FFP) were mostly( 75%) allergic in nature.

*Conclusion:* FNHTRs are increasingly getting reported due to the fact that no premedication is being given routinely and increased awareness of the physicians about the hazards of blood transfusion. Prestorage leukoreduction of blood components and warming the blood using inline warmers may be a viable option to prevent FNHTR.

#### Seroprevalence of cytomegalovirus among voluntary blood donors

**C. N. Chaudhari**

INHS Jeevanti, Goa Naval Area, Vasco-da-gama Goa, India

*Background:* Primary Cytomegalovirus (CMV) infection in immunocompetent host is self limiting infection, leading to latency of virus. However congenital CMV and CMV infections in immunocompromised patients is associated with high morbidity and mortality. Transfusion Transmitted – Cytomegalovirus (TT-CMV) infection in low birth weight neonate and immunocompromised transfusion recipients is being increasingly reported. Studies recommended transfusion of CMV free or CMV safe blood in prevention of TT-CMV. In this background, the study was undertaken to know the CMV seroprevalence in blood donor.

*Materials and Methods:* Prospective study was conducted. 431 voluntary blood donors were screened for CMV IgG and IgM by EI A (Enzyme Immuno Assay).

*Results:* A total of 379(87.9%) voluntary blood donors were seropositive for CMV IgG. There was no statistical difference of CMV seropositivity and age. Further, 7(1.6%) subjects were both CMV IgM and IgG seropositive.

*Conclusion:* High seroprevalence of CMV in our donor population is threat to the blood safety. Strategies in reducing the risk of TT-CMV discussed. Use of prestorage leucodepted ‘CMV safe’ blood components along with judicious use of blood is recommended in prevention of TT-CMV in high risk recipients.

### Blood donations and apheresis

#### Standardization of RBC units for HB content based transfusion policy

**J. Mathai, P. N. Sindhu, P. V. Sulochana, S. Sathyabhama, S. Arun kumar**

Blood Transfusion Services, SreeChitra Tirunal Institute for Medical Sciences andTechnology, Trivandrum, India

*Background:* Other than specifying the volume of blood to be collected, current standards do not address the Hb content of a rbc unit after processing, resulting in poorly standardized units. Individual units of rbc can vary widely in Hb content due to differences in donor Hb concentration, collection volume and preparation losses. Transfusing physician often finds a poor correlation between number of units transfused and recipient's increase in Hb. In order to reduce this variation, there is need to standardize donor units for predicting benefits of transfusion.

Aims:

1. Standardize rbc units with an Hb content of 50± 5 gm/bag,
2. Evaluate Hb level achieved with a standardized unit in adult anemic patients. Methodology: Blood was collected from healthy blood donors in 450 ml double bags for component separation. EDTA sample was obtained along with collection and donor Hb level was estimated. Standardization of RBC units was achieved by adjusting volume depending on donor's Hb concentration. Hb gm/bag was assessed from donor Hb gm % and volume of RBC. Patient demographic details, no. of transfusions, age of units and pre and post transfusion Hb levels were collected. Meant ± SD was calculated for the variables.

*Results:* These standardized rbcs units were grouped according to their Hb content. Out of the units available under study, 60 units were transfused for 52 patients with a mean post transfusion Hb increase of 2 gm%. Nearly 87% of transfusion orders were met with one unit of rbc with a mean Hb level of 8.5gm/dl.

*Conclusion:* A protocol was evolved for having a standardized unit of rbc with acceptable Hb content/bag. This should be a high priority goal for Blood Centers as knowledge of actual Hb content can be used to optimize rbc transfusions, limiting donor exposures and providing therapeutic dosage.

#### Quality enhancement of plasma and yield by contact shock freezing

**P. S. Rao, S. R. Joshi, R. B. Sawant**

*Background:* Fresh frozen plasma (FFP) is exposed to numerous factors affecting the quality and stability of coagulation during blood collection, preparation and storage with guidelines for GMP and of FDA for quality blood components are –

(A) Temperature (B) Time. Components are always manufactured with in the said time and temperature as per the International standardsand plasma's are frozen at -800 C, approximately takes 6-8 hrs, only if the deep freezer doors are not opened frequently with no fluctuation in current and with minimum load Method Trial study was carried out using Dometic contact shock freezer (CSF). The temperature of - 400 C to the core is reached within 35 to 40 minutes. Actual time taken for freezing plasma was just 30 minutes for 25 plasma units (depends on plasma volume). These frozen “plasma cakes” were shifted to - 400 C frost- free storage freezer.

*Result :*

1. Contact Shock freezing to the core temperature of plasma ≤ 400 C with most low temperature deep freezing technology optimized cooling system within no time was obtained.
2. Factor VIII activity of randomly prepared FFP at -800 C was 70 - 80%. which was enhanced with CSF (120 -130%) the preservation of factor VIII was 50 % more than our routine procedure.(P. < 0.01)
3. QC of cryoprecipitate revealed, factor VIII levels 50% more (90 – 132%,*P.* 0.01). However for Fibrinogen it was almost 75% more (2.9 - 4.2*P.* <0.001)
4. Routine plasma freezing procedure we get amoeboid shaped bags (Indian papad)results in leakage of plasma bags and consuming more space due to uneven shape of the bag. As compared to that of plasmas prepared with CSF uniform and “flat“ regular shaped bags (Just like a piece of Cake), deep freezer space utilization is significantly more and wastage of 10 – 20% is completely saved.(either keeping the bags upright / stacked due thin and 100% flat surface) Plasma segment were straight and flatly frozen.
5. Electricity consumption is significantly less (2 hrs. / 75 plasma units) with good air conditioning in component manufacturing room than that of routine freezing (- 800 C continuously working for 24/7, 365 days), which in return a monitory benefit to the Institute.

Conclusions:

1. CSF is user friendly and hassles free, reduces work time with a significant yield of factor VIII and fibrinogen levels in Cryoprecipitate and factor VIII levels in FFP than conventional technique.
2. Wastage of plasma is dramatically reduced, 100% utilization of space in storage freezer. Moreover the operating and running cost benefits the end-user.
3. With such simple and well tried scientific principles of high quality plasma leading to high quality derivatives can be assured.
4. This Contact Shock freezing equipment will be a boon in field of Transfusion Medicine in India

#### Unusual events during platelet donation using the latest apheresis systems

**N. Agarwal, S. Ojha, R. K. Chaudhary, A. Verma, A.Sonkar**

Department of Transfusion Medicine, SGPGIMS, Lucknow, India

*Background:* Plateletpheresis are considered safe however adverse events related to these procedures have been discussed elaborately in the literature. Most donors suffer a mild reaction rarely requiring any medical intervention. Citrate-related reactions are the most common and comprise of 30-40% of all apheresis donor reactions. We at our apheresis unit frequently encounter various donor reactions most of which can be managed by our apheresis team, only few requiring some medical or emergency attention. Here we presented our experience of encountering and managing donor reactions caused by plateletpheresis.

*Materials and Methods:* The prospective study included 373 plateletpheresis procedures performed using 3 latest apheresis machines on eligible donors over a period from October 2007 to June 2008. All procedures were performed following the departmental standard operating procedure (SOP). Procedure details were explained to each donor and all were directed to report any form of discomfort or uneasiness to the apheresis team immediately. Details of donor reaction physiologic or adverse and their management were documented in the procedure register.

*Results:* Mean pre-apheresis platelet count of the donor population was 207.8 × 106 /ml with a mean Hb value of 13.9 gm/dl. A total of 50 prospective donors had undergone donor reaction of which 39 (79%) had citrate related reactions with a female preponderance. Donor reactions were observed in 28% females and 12.7% males. Of the citrate related reactions 30 experienced paresthesias, 7 had muscle cramps with nausea; vomiting and 2 donors had severe reactions in the form of tetany and carpo-pedal spasm. Donor reaction were significantly higher in younger (<25years) and less weight (<60 kg) donors (*P* < 0.05).

*Conclusion:* Although donor reactions due to plateletpheresis are generally transient and self-limiting, these should be identified immediately and managed accordingly. Donor motivation and close observation during plateletpheresis can reduce such incidences significantly. Proper donor care helps in retention of such donors and enables maintaining an effective donor registry.

#### Evaluation of hemolytic disease of newborn in artificial model using anti-d and anti fetal hemoglobin antibody by flow cytometry

**Abbasali Pourazar, Homayouni Vida, Rezaei Abbas, Andalib Alireza, Oreizi Farzad**

Department of Immunology, Isfahan Medical School, Isfahan University of Medical Sciences, Isfahan, Iran, India

*Background:* When fetal red cells, enter the maternal circulation from placenta an event would be happened that is described as feto-maternal hemorrhage (FMH). This life threatening condition could be detected by using RBC antigens (surface antigens and intracellular antigens). Therefore the measurement of fetal RBCs in an artificial model would be useful to calculate of FMH and consequently the dosage of Rhogam for prophylaxis.

*Objective:* Evaluation of the feto-maternal hemorrhage in an artificial mixture model.

*Materials and Methods:* A series of 40 artificial specimens were prepared consisting of Rh(D) negative adult blood (none immunized) spiked with varying amounts of Rh(D) Positive cord blood (from mothers between 20-30 years old in Shahid Beheshti Hospital). Monoclonal anti-D and anti-HbF staining for detection of the fetal RBCs in artificial mixture sample modeling were used.

*Results:* This study showed that the percentage of fetal cells in the artificial sample for anti −D antigen is in ranges of 0.28% - 0.32% for a 0.25% dilution mixture, and 1.3% - 2.05% for the mixture with dilution 2%. In addition, the ranges of data for anti HbF staining was obtained 0.2%-0.34% for the 0.25% dilution sample, and the ranges of 1.04 -1.8% for the 2% dilution. The regression analysis indicated that the correlation of anti-D assessment with expected standard method was r2=0.9672 and anti HbF assessment was r2=0.8842.

*Conclusion:* Although both molecule targets could be used for detection of fetal RBC, in this model, anti -D staining was more accurate than anti- HbF staining. However, since anti-D can not be utilized for low-density or weak phenotype and other incompatibility, the anti HbF labeling could be used for all feto-maternal hemorrhage.

#### Neonatal transfusion practice

**K. C. Usha**

Department of Transfusion Medicine, Medical College. Thiruvananthapuram, India

*Background:* Child is not a miniature of adult and neonate is not a miniature of child. Various aspects of Transfusion medicine are unique to neonates such as neonatal physiology, small size of neonates, neonatal immunology, standards of compatibility testing, neonatal vascular access and indications where transfusion support is required. Newborn responds to anaemia with poor cardiovascular response and poor and delayed bone marrow response. Blood / components should be brought to room temperature before transfusion. Otherwise hypothermia can make a new born sick especially preterm. This is more so for exchange transfusion where large volumes are used. Newborn poorly tolerates calcium, potassium and lactate loads. It is better to use blood <5 days old. Similarly old blood with depleted 2 -3 DPG will further shift oxygen dissociation curve to left, which is already to left due to HbF. Small size of the new born makes the requirements of blood / components in small volume.

New born particularly premature ones with birth weight <1.3 kg require multiple RBC transfusions during 1st few weeks of life. Associated infections and repeated blood samplings for laboratory investigations may lead to anemia. During later weeks, inability to mount an effective erythropoietin response to falling RBC value (Haematocrit /Hb) may lead to additional transfusions. Anemia remains a major problem for many premature infants. During the 1st week of life fall in haematocrit or Hb level, may be either physiological or pathological. In healthy term neonates Hb rarely falls below 9g/dL at approximately 10 – 12 weeks of age. This decline in Hb is earlier and more pronounced in premature infants, even in those without complicating illnesses.

The lower Hb values in premature infants than those of term infants are due to decreased erythropoietin output in response to anemia. There is inadequate production of erythropoietin rather than abnormal response of erythroid progenitors to growth factor. Transfusion is advocated to keep haematocrit >40% during severe cardiopulmonary disease and >30% for major surgeries.

*Neonatalimm unology and compatibility testing:* Both cellular and humoral immunity is immature in a new born. Almost all of the antibodies present in newborns are of IgG class and are derived from mother transplacentally. Hence standards of pre transfusion compatibility testing can be relaxed for first 4 months of life. Donor blood and baby's blood tested for ABO and Rh groups. Mother's blood is tested for ABO and Rh groups as well as for antibody screen. If mother's blood is not available baby's blood is tested for Antibody screen. If Antibody screen is negative subsequent transfusions can be given without cross match provided the blood is of either O group or is ABO identified with both child and mother and is Rh D negative or Rh D compatible with child. If Antibody screen is positive, corresponding Antigen negative blood should be given as far as the antibody persists in neonatal circulation. Immature cellular immunity especially in preterm leads to increased chance for TAGVHD Hence irradiated blood /products advocated. In neonates blood / products may be given as small aliquots.

A) Whole Blood

The indications for whole blood transfusion is very minimal. Main indication is exchange transfusion and blood less than 5-7 days old should be used. 15 cc /kg of whole blood will raise Hb by lgm%. Other indications in newborns are massive hemorrhage, cardiac surgery etc.

Single / double volume exchange transfusions may be required to treat Rh or ABO in compatibility. While using O group those with low anti A and anti B titer should be selected.

*B) Components* 

When blood is given as components harmful effects of whole blood transfusion such as plasma overload, lymphocyte mediated toxicities; allosensitisation etc can be prevented to a certain extent.

*PRBC:* Both volume as well as O2 Carrying capacity can be replaced by PRBC. Ideal Haematocrit for PRBC is 70-75%. This product is useful for neonatal anemia including acute blood loss provided there is no massive hemorrhage / shock. 15 cc /kg of PRBC will raise Hb by 3gm%. In a very sick pale neonate with CCF, it is better to do a partial exchange transfusion to correct HCT as it will not lead to sudden volume overload.

*Platelets:* ABO /Rh identical/compatible donors may be selected for platelet preparations. There will be enough stroma of Rh D positive cells in the platelet preparations, sufficient to lead to Rh sensitization. If platelets from Rh +ve donors are infused to Rh -ve individual, Rh immunoglobulin prophylaxis should be given. One should use low Anti A / Ant iB antibody titer donors whenever possible. Even the little amount of plasma with high titers of Anti A /Anti B present in platelet preparation is sufficient to produce significant haemolysis in newborn. Random Donor Platelets (RDP) or Single Donor Platelets (SDP) can be used. 10 ml/ kg of platelets from RDP will raise platelet count to 75000 to 100000mm^3^. RDP is less costly and easily available from blood bank. SDP is a platelet concentrate obtained by apheresis. It has 6 – 7 times more platelets than RDP. It is more concentrated and more effective. SDP can be divided into 3 to 4 units for use in so many babies or repeatedly for same baby. CMV negative, HLA matched donor may be selected.

Indication for platelet transfusions in newborn may be prophylactic/therapeutic.

*Granulocyte preparations:* Help to decrease mortality due to sepsis. Many a times, a sick neonate has neutropaenia/or neutrophil dysfunction or both. Hence it is logical to use granulocyte transfusions in septic newborns especially if they are not improving with antibiotics and antifungals. With the availability of colony stimulating factors, granulocyte preparations are rarely used nowadays. The best way is to prepare by apheresis. Recommended dose is 1-2×109 granulocytes/kg body weight in 10 -15 ml of plasma. This may be repeated every 12 – 24 hours for 4 – 6 days. One unit obtained by apheresis will have 1011 granulocytes in 200ml of plasma. Those from buffy coat have 109 in 100- 200ml of plasma. The donor should be ABO/Rh compatible and CMV negative. Advisable to irradiate the unit before transfusion especially in preterm to prevent GVHD, as these packs may contain plenty of lymphocytes. They are stored at room temperature. Shelf life is 24 hours.

*Lencodepleted blood components:* Antibodies can develop against lymphocytes and lead to FNHTR. Lymphocytes lead to allosensitisation. If not leucodepleted, graft rejection occurs in prospective candidates for BM transplantation. Lymphocytes can act as carriers of HIV, HTLV1, EBV, CMV etc. In newborns transmission of CMV and transfusion associated with GVHD are the most important complications related to donor lymphocytes. Fresh frozen plasma: Contains both stable and labile coagulation factors. 1 ml of FFP contains approximately 1 unit of each clotting factor. Dosage of FTP is 10 -15 ml/kg every 12 hrs. Indications are haemorrhagic disease in newborn. DIC, Liver disease with coagulopathy, inherited factor deficiency etc.

*Cryoprecipitate:* Very rich in factor VIII and XIII, fibrinogen, von Willibrandsfactor Dosage is 5 cc /kg every 12-24 hrs. Indications are haemophiliaA with bleeding, DIC, vWB factor deficiency etc.

*Conclusion:* For survival, the low birth weight and very low birth weight babies need a prolonged stay in ICU with intensive monitoring and supportive care with blood / components. Judicious use of blood components in such babies is beneficial and life saving. Use of correct component helps prevent wastage of blood and makes it safer for newborns. The outcome of sick neonates especially preterm has been greatly improved by the availability of better and safe blood components.

#### Thrombophlebitis of antecubital vein: A rare sequel of donor phlebotomy

**S. Sachdev, R. Kaur, P. Sinha, G. Kaur, P. Kaur, S. Basu**

Department of Transfusion Medicine Government Medical College and Hospital, Chandigarh, India

*Background:* Non systemic local reactions among blood donors are less common than the vasovagal type. For this reason alone it is important to recognize such events, and identify the factors contributing to the occurrence of such a rare event. Incidence of post phlebotomy thrombophlebitis is .0001% as per literature.

*Materials and Methods:* A donor presented with feature of thrombophlebitis after blood donation (first in 89000 donations at our centre since inception). A detailed donor examination and history along with an assessment of donor records was undertaken. An underlying simmering eczematous condition of the skin was elicited. History of reaction among patients who received the blood components from this donor was assessed retrospectively.

*Results:* Donor was treated, counseled and motivated for future donations. A review of the deferral for skin disorders was undertaken and the same was revised.

*Conclusion:* Donor deferral due to chronic skin disorder might need to be reevaluated. Importance of proper sterilization of cubital area is reemphasized. Donor screening is a very important step for safe blood transfusion.

#### Donor notification and counseling: Experience at our blood center

**R. Kaur, G. Kaur, P. Kaur, S. Basu**

Dept of Transfusion Medicine, Govt. Medical College and Hospital Chandigarh, India

*Background:* Advising blood donors who have positive viral marker test results is an essential adjunct to blood donor testing. Informing donors of positive results provide them with information about follow up diagnostic evaluation, currently available medical and psychosocial services and assists in preventing transmission to others. As a part of Blood Safety programme, NACO Ministry of Health and Family Welfare, Government of India gave guidelines for revealing the TTI status of the donor after proper counseling and taking informed consent. We present experience with donor notification and counseling at our blood bank.

*Aim:* To evaluate the effect of implementing blood donor counseling programme in our set up.

*Materials and Methods:* At our Blood Centre all voluntary and replacement donors are being notified since September 1,2004. All donors are counseled regarding TTI, their mode of transmission, mandatory testing and given option to be personally informed about reports of testing done for diseases. The reactive donors who give consent are informed through letters. During revisit the test result is revealed to the donor and a repeat sample of the donor is taken after performing post donation counseling. Confidentiality is ensured during the entire procedure.

*Results:* A total of 18,889 donors were counseled during the study period (Jan 1, 2005 to June 30, 2008).A small number of reactive donors (22%) actually returned for post donation counseling. Majority of these donors were male donors and belonged to 20-30 years of age group and were seroreactive for HBV, HCV, Syphilis. Only two HIV reactive donors reported back for post donation counseling. Problems faced were:

1. Donors denying the consent to be informed about their TTI status
2. Wrong/incomplete address mentioned on the donor cards
3. False positive and indeterminate test results
4. Non availability of trained counselor

*Conclusion:* Counseling regarding TTI is essential and effective strategy to achieve a safe source of blood supply. However, the procedure is immensely complex. An improved system for donor follow up and availability of trained counselor is required. Factors leading to decreased donor return rates for post donation counseling needs to be evaluated

#### Developing an algorithm for mass screening of IDA and BTT in blood donors

**Tiwari AK, Chandola I, Alok Ahuja**

IMA blood Bank, 47, Ballupur Road, Dehradun, India

*Background:* IDA and BTT are the most common causes of microcytic anemia in blood donors. However, the prevalence of microcytosis in blood donors and prevalence of IDA and BTT in microcytic donors has not been studied. Blood banks could also contribute in diagnosing these two conditions, provide counseling and/or treatment.

*Objective:* To determine the prevalence of IDA and BTT in blood donors and develop an algorithm for mass screening.

*Materials and Method:* A total of 925 donor samples were evaluated on a calibrated cell-counter. Of these, 50 were microcytic. These 50 microcytic and 50 age and sex matched normocytic donor samples were subjected to plasma ferritin and quantitative determination of hemoglobin types to establish diagnosis of either IDA/BTT.

*Results:* The prevalence of microcytosis was found to be 5.4%. Amongst the microcytic donors, 52% were diagnosed with IDA, 36% with BTT, 8% with both and 4% had none. In case of normocytic donors 26% were diagnosed with IDA, 6% with BTT and 4% had both.

*Conclusions:* The number of IDA in the microcytic samples was found to be twice that of normocytic samples while in case of BTT, it was 6 times that of normocytic samples. The algorithm therefore would be to do hemogram on all donor samples, routinely. Plasma Ferritin should be done on all microcytic (only 5.4 % of all donors) samples. If the level is lower than 15 ng /ml, it is diagnosed as IDA. If the levels are higher than 15 ng /ml, HPLC tests are done to diagnose or exclude BTT. By employing this algorithm majority of IDA and BTT can be diagnosed while keeping the number of Ferritin tests small and number of HPLC tests even smaller and thus making it cost effective.

#### To study the effectiveness of different modes of communication for retrieval of temporarily deferred donors

**R. Shah, S. Tulsiani, U. Chudgar, N. Choudhury, P. Desai, R. Jain, V. Harimoorthy, A. Mathur**

Prathama Blood Centre, Ahmedabad, India

*Background:* To enhance safety of blood units, strict donor selection criteria are followed which leads to deferral of many motivated donors. Efforts should be made for retrieval of the donors who are deferred for temporary reasons.

*Aim:* The present study was carried out to evaluate the mode of communication best suitable to contact donor for donation after temporary deferral and the time taken for donor response.

*Materials and Methods:* During the study period of 12 months (August 2006 to July 2007), 34625 donors donated whole blood altruistically at Prathama Blood Centre, Ahmedabad. Number of donors deferred was 10896 (33%). From this group, randomly selected 1000 temporarily deferred donors were included in the study. At the time of deferral, donors were asked about their preferred mode of communication (personal phone call, letter and e-mail) and were called accordingly. The time taken for response was also evaluated by dividing into 3 groups: Immediate (< 7 days), Intermediate (7 days to 1 month) and delayed (> 1 month).

*Results:* In the follow up of these 1000 deferred donors, no. of donors called by phone were 852 (85.2%), by letter 127 (12.7%) and by e-mail 17 (1.7%). 4 donors (0.4%) came on their own before their due date of re-call (self). On retrieval, 312/1000 (31.2%) donors came back to donate blood. Percentages of donors who responded to phone calls were 284/852 (33%); letters were 22/127 (17%); e-mails were 2/17 (11%). Out of these, 270 were accepted for donation and the time taken by these donors for response was also studied. Immediate response was noted in 131/270 (48.5%); intermediate response in 77/270 (28.5%); and delayed in 62/270 (23%).

*Conclusion:* Motivated donors who are temporarily deferred are willing to come again for donation if they are contacted. Personal telephonic contact was the more effective mode of communication for retrieving donors. Blood centre should develop a mode of recall for temporary deferred altruistic donors and try to retain them.

#### Challenges in recruitment and retention of voluntary blood donors- an experience of two decades

**Blood Kumar**

Voluntary Blood Donors Organization, Chennai, India

*Background:* The three basic aims of Blood Transfusion Services (B.T.S) are safety quality of blood transfusion and last but not least the adequacy of blood supply. These aims can only be achieved from Non- Remunerated and truly altruistic blood donors from the low risk populations. There are lots of challenges in recruiting and moreover to retain them and these challenges become of particular importance in developing countries like India where literacy rate is relatively low and there are lot of associated myths related to blood donations. To motivate and recruit the blood donors we prepared a set of well designed educational material for blood donors’ recruitment. In poor resource settings to develop and implement programme to promote Voluntary blood donation required lot of skill so that the message reaches to the maximum number of people with long sustainability.

*Materials and Methods:* To motive, recruit and retain the blood donors we used posters with appealing slogans in local languages. Furthermore, we used audiovisuals of some roadside victims, stampede and other mass casualties in which we highlighted how the blood saved lives of victims. We took help of voluntary repeated donors and the leaders from the local community to motivate them.

*Results:* motivation of donors is directly proportional to the literacy rate of the population. Some got motivated in single visit and for last 20 years they are the regular donor some are so stubborn and attached to unknown myths that still we are trying to motivate them.

*Conclusion:* To get the truly altruistic voluntary donors the first and foremost requirement from the motivator side that they should also be truly altruistic in their approach. If motivator is not involved fully the major problem faced is the retention of blood donors.

#### Donor retention: Futuristic approach

**L K. Dadhich, V. P. Gupta**

ISBTI, Kota, Rajasthan, India

Experiences of the past for donor retention have been useful for ISBTI to develop futuristic approach with an ageing population as seen in India the Blood Transfusion Service (BTS ) estimating blood demand increase by 27% in next 20-25 years. Therefore, continued effort should be made to maintain the donor pool sufficient to meet the local blood demand. But, donor recruitment and donor retention is not an easy task in blood service because of the dynamic nature of the socio-economic environment and the human factor involved. While the effectiveness of recruitment and retention is usually determined by the degree of adequacy in blood supply. Some qualitative but important issues such as donor satisfaction and loyalty are frequently overlooked. It is well known and recognized fact that there always exists a group of otherwise healthy adult refuse to come forward for blood donation.

Donor recruitment drives involve knowing local demography, local communities, their thinking, living standards, socio-economic patterns, leisure activities, local role models, heroes and heroines, and then targeting the people who are most likely to become safe regular donors in a cost effective manner.

Successful donor retention depend on experience of donating blood first time, how comfortable and convenient it has been, how efficiently the records are kept, recalling of donors, reminding them how much they are needed, thanking them for their contribution and assuring them proper utilization of their donated blood. Looking to the above extra effort should be put on, to develop good recruitment strategies, that are culturally sound and socially acceptable to the population concerned. Without such a mean to keep the blood donor pool growing positively, it will incur definite risk to the adequacy in the blood supply and hence, endanger patients care. Therefore, the blood service should develop appropriate strategy in blood donor retention.

The paper highlights systematic and futuristic approach on donor recruitment and retention and discusses strategies to be employed in donor retention.

#### Donor management

**S. Padma**

Government General Hospital, Chennai, India

Donor Management 1. Pre Donation 2. Post Donation Pre Donation: 1. Treated as VVIP 2. Given First Attention, Counseling 3. Blood Donation Form writing Post Donation 1. After Blood Donation?” Refreshments like biscuits, Juice 200ml, Mineral water 500ml, to replace 350ml Blood taken. 2. B.D Certificates, grouping cards are given to them immediately 3. Any untoward reactions after BD are very rare. They may be as follows: The nature of treatment of all adverse reactions should be noted in Donors record. Donor Recruitment: 1 Donors should be recognized and treated as WIPS 2 Donors are encouraged to donate blood once in 3 months to become a voluntary, regular safe donors 3 College students because of awareness and motivation activities have become regular donors 4 Counseling is done to assure them of no complications after donations 5 Donors are told about the component preparation, it uses and about the results of the mandatory tests with their consent about informing them, which helps in motivating them. Donor Retention 1. Motivation and Awareness helps in retention of voluntary safe donors. 2. Voluntary, Non-remunerated Donors are considered safer than others. 3. They should be assured about the confidentiality about their mandatory test results and is informed to them only with their consent. 4. Donor retention form, Greetings, Feedback forms are given and said about the importance given by them which will be executed i.e. their ideas and values 5. Regular voluntary Donors should be honored on special occasions like: world blood donors day: National Voluntary Blood Donors day. If possible the Governor or the highest official of the concerned place should give them awards, prizes.

#### An analysis of motivational factors in blood donation- a tool for targeted blood donor recruitment

**U. R. Subramanyan**

Department of Blood services, Ministry of Health, Oman

*Introduction:* The blood donor population in Oman consists of voluntary (65%) and replacement donors (35%). Identification of the underlying motivational factors in donors is crucial to apply proper recruiting strategies. This study is designed to identify demographic, attitudinal and influencing factors, and to assess the importance of each factor in blood donation. The results can be applied to any developing country with similar donor groups.

*Materials and Methods:* Data from a questionnaire survey of 116 randomly selected donors were used to evaluate demographic, attitudinal and influencing factors in a fixed collection site. The data about the donor s preferred mode of reminder and his donation experiences were also analysed. The relation of these factors to the donor status (first time vs. repeat) was examined.

*Results:* Many donors identified more than one reason to donate. Donors who have donated more than 5 times are ten times more likely to state ”right thing to do” (altruism) as the reason to donate than the donors who have donated less frequently (OR 10.21, CI 3.07–33.94). Donors with secondary school education or below were at least twice as likely to be donating for family reasons compared to more educated donors (OR 2.34, CI 1.05-5.24). The odds of the first time donor being a family donor were 2.67 times higher than the repeat donor (OR 2.67, CI 1.06-6.70). Less educated donors were more likely to donate because of being asked by a recruiter (OR 6.70, CI 1.02-43.9).

*Conclusion:*

1. Altruism is the chief factor and is more common in repeat donors who are aware and sensitized already. Stressing on the emotional satisfaction from fulfillment of civic or human duty can play a vital role in blood donation.
2. Less educated replacement donors if properly recruited and retained, can add to the existing voluntary donor pool. Non monetary incentives, reminders and positive donation experience will bring them back for blood donation. Studies done in India are compared and relevant points will be presented.

#### Comparison of infectious disease markers in voluntary and replacement blood donors at Bhabha Atomic Research Centre hospital in Mumbai

**V. Arora, R. K. Kulkarni, S. Cherian, R. Pillai**

Bhabha Atomic Research Centre Hospital, Mumbai, India

*Background:* Blood and blood products are a potential source of transmission of Hepatitis B and Hepatitis C viruses, Human Immune deficiency Virus (HIV) infection and Syphilis. Blood safety remains an issue of major concern in transfusion medicine. The objective of this study was to determine the frequency of these infections in voluntary and replacement donors at BARC hospital blood bank.

*Materials and Method:* Consecutive samples of 3368 apparently healthy donors were tested for HIV, Hepatitis B, Hepatitis C and Syphilis over a period of six years i.e. 2002 to 2007. Out of these, 15.32% (516) were voluntary donors (non-remunerated) and 84.68% (2852) were replacement donors. Our replacement donors are relatives and colleagues of patients who willingly came to donate blood after their patient has been transfused blood. Seroprevalence of infectious markers were compared between the two groups using standard statistical methods.

*Result:*2.11% (71 donors tested positive) of the donated blood had serological evidence of infection with one of the pathogens tested. Seroprevalence in voluntary donors was 0.97% (5 voluntary donors tested positive) and that in replacement donors was 2.31% (66 replacement donors tested positive). This difference in seroprevalence is statistically significant.

*Conclusion:* Higher prevalence of HIV, HBV, HCV and Syphilis are observed in replacement donors. Hence, voluntary blood donation is advocated as it has been observed to be safer.

#### Hypertension: A factor for deferral in donor screening

**A. Bhattacharjee, T. Chaliha, M. Sarma**

Department of Transfusion Medicine, Guwahati Medical College and Hospital, Guwahati, India

*Background:* Blood donation is one of the noblest human services. A casual approach to donor selection can lead to certain complications. Every effort was made from time to time to obtain blood proper for donation. While taking blood one should meticulously verify that the blood taken from the donor does not have any adverse effect either on the donor or the recipient. Aim: To study the prevalence of hypertension among blood donors.

*Materials and Methods:* A prospective study was conducted in the Dept. of Transfusion Medicine, Gauhati Medical College and Hospital between Aug’2007 and July’2008. Total blood collection was 19,879 units.(Male-18,980 and Female-899). M: F ratio was 21:1. Out of these 18,472 were replacement donors and 1407 were voluntary donors. Ratio of replacement to voluntary donors was 13:1.

*Result:* A detailed history of pre-existing problems like pulse and blood pressure recordings was done. Out of the total collection of 19,879, 540 (2.72%) were deferred. The main causes of deferral were due to high blood pressure IN 142(26%) cases, 66 (12.2%) cases for low hemoglobin, 40(7.4%) cases for self-inflicted cut marks, 50(9.2% )for underweight and 60(11.1%) for skin infection. 30(5.56%) were professional donors. 22 (4%) donors has been deferred for tattoo mark), 14(2.6%)gave history of malaria ,and rest for other causes like underage, history of of asthma, epilepsy and tuberculosis etc. Out of the 142 deferred due to hypertension 132(93%) were replacement donors and 10 were voluntary donors, amongst these male donors were 126 and 16 were female donors (M: F 8:1). The age ranged from 18yrs to 20yrs -12, 21yrs to 30yrs-25, 31yrs to 40yrs -40, 41yrs to 50yrs -47, and 51yrs to 60yrs -18.

*Conclusion:* There exists a difference of opinion between the Drugs and Cosmetics Act and National Aids Control Organization (NACO) guidelines regarding acceptance of blood from hypertensive donors. NACO considers that it is safe to take blood from hypertensive donor once the blood pressure is well controlled by medications. But this is not incorporated in the Drugs and Cosmetics Act of India till date. As such it is legally unsafe to take blood from a donor who is hypertensive and medical personal should be extra careful in donor selection which is having legal implications.

#### Unusual events during whole blood donation: SGPGI experience

**S. Ojha, N. Agarwal, A. Verma, A. Sonker, R. K. Chaudhry**

Department of Transfusion Medicine, SGPGIMS, Lucknow, India

*Background:* Withdrawal of a unit of whole blood is usually well tolerated in healthy adults, but occasionally a donor may experience an adverse donor reaction. These reactions vary considerably in nature and severity and can negatively impact blood donor return rate.

*Aims:*

1. To find out the prevalence of unusual events during whole blood donation
2. To study association of various factors with probability of donor reaction
3. Objective assessment of reactions in the form of laboratory parameters Material and methods: Retrospective analysis of factors which may predict outcome of donor reactions was done for a period of 3 years from January 2005 to December 2007. Odds Ratio was used to measure strength of association between risk factor and outcome in reactors vis-à-vis non-reactors. Objective assessment of donor reaction was done by measurement of serum cortisol in first time, repeat and reactor donors.

*Results:* In 57603 whole blood donation, reaction rate was 1.8%. Reaction rate was higher in female donors(2.9%) compared to male donors(1.7%), but gender per se was not predictor of donor reaction. Majority(85%) of reactions were mild in nature. Young donors (<25 years) had higher chances of reaction(2.6%).However body weight had no bearing on reaction rate. Similarly first time donors were more prone for donor reaction(2%).Modifiable factors like longer predonation waiting period, donation around noon and food intake>4 hours also increased risk of donor reaction. Serum cortisol was higher in reactors.

*Conclusion:* Registration of all unusual events helps to improve donor safety in a rational way in the form of careful history to minimize modifiable factors of donor reaction and appropriate intra and post donation care.

#### Demographic risk factors associated with undetected hypertension in helathy blood donors of Kerala

**J. Mathai, R. Nair^1^, P. V. Sulochana, S. Sathyabhama**

Blood Transfusion Services and ^1^Cellular and Molecular Cardiology, Sree Chitra Tirunal Institute for Medical Sciences and Technology, Trivandrum, India

*Background:* Hypertension usually does not have any warning signs and has been called the ‘silent killer’ as it is a predisposing factor for cardiovascular morbidity and mortality. Analyzing the causes of donor deferral over a period of three years (2004-06), large fraction of healthy blood donors (24.4%) were rejected on the basis of hypertension. Apart from losing donors, this has a negative impact on their future donor return rates. In addition, the sequel of hypertension poses a public health problem.

Aims:

1) Determine the proportion of individuals with undetected hypertension 2) Identify demographic risk factors associated with hypertension Methods: Individuals who reported to the Blood Centre for voluntary blood donation constituted the study population. Deferred hypertensive individuals (study group) were compared with age and sex matched accepted donor controls. As a pilot study, 50 hypertensives were compared with 100 unaffected controls. Donor was administered a pre tested structured questionnaire, which included information on demographic variables, medical history and information on lifestyle. At the time of health check, the medical officer measured the BP and hypertension status was assessed on the criteria of WHO-ISH and US Seventh Joint National Committee Report. The data was statistically analyzed using SPSS package.

*Results:*1% of healthy donors had undetected hypertension. Features that showed significant association with hypertension were higher BMI (OR 4.71) and positive family history (OR 2.325).

*Conclusion:* Early detection of hypertension with possible risk factors will help in planning interventional programs including dissemination of information on life style modifications. The prevalence of hypertension in the apparently healthy individuals warrants further studies to offer prompt management and prevention of unfavorable squeal. Monitoring health parameters of donors will expand the perception of a blood bank from a center of transfusion services to a place of comprehensive individual and community health.

#### How yoga can achieve 100% voluntary non-remunerated blood donation in india

**T. R. Raina**

Blood Bank, SMGS Hospital and Govt. Medical College, Jammu, India

Human blood is a vital fluid constantly pumped by the heart throughout the body and consists of Red Blood Cells, White Blood Cells, Platelets, Plasma, Proteins and other elements. The composition of blood is so complicated that it cannot be manufactured artificially and the only source of blood is human body. Human blood is the most precious entity gifted by the God to the mankind and there is no absolute substitute for human blood. In fact blood is the life force circulating in the body in the form of liquid love. Safe blood saves life but at the same time contaminated blood can take life. Blood Transfusion services in our country have gained special significance in the recent years and form a vital part of National Health Care System. Out of all types of Donations, Blood Donation is the most sacred and pious act and Voluntary Non-Remunerated Blood Donation (VNRBD) is the safest among all Blood Donations. Many modes and methods have been devised and practiced to motivate, recruit and retain Voluntary Blood Donors but 100% Voluntary Blood Donation as per the Action Plan of National Blood Policy by December 2005 has not been achieved yet. Yoga is an ancient method of uniting body, mind and soul based on scientific foundations. There are different methods of Yoga and how yoga shall help in achieving the action plan of National Blood Policy i.e. 100% Voluntary Blood donation shall be discussed in detail.

### Components

#### Standardization of procedures in umbilical cord stem cell processing and storage

**T. Chandra, A. Kumar, A. Sharma, U. Singh, M. S. Kushwaha**

Department of Pathology and Blood Bank CSMMU, Lucknow, India

*Aim:* Umbilical cord stem cells are being used for the treatment of various malignant and non malignant diseases and research on them will help to change the treatment of various diseases. Aim of the study was to standardize the procedures of umbilical cord stem cell collection, processing and storage in KGMU.

*Materials and Methods:* Total 50 samples of umbilical cord blood were collected from donor mothers who were full term healthy pregnant females. Various techniques of collection, processing and storage were compared and modified to give the maximum yield of mononuclear cells.

*Results:* Maximum average volume of umbilical cord blood was collected when placenta was in utero. An average mononuclear cell count of 6.23±2.76xl07/ml was obtained. Optimum yield of mononuclear cell was obtained by double centrifugation tube method. The mean MNC count changed from 7.37xl07/ml to 6.9xl07/ml with average percentage lose of 7% mononuclear cell. Optimum storage was seen with DMSO at −70° c with viability of 78.89%, 75.69%, 74.19% and 67.26% at just after processing, 1month, 2months and 6months intervals respectively.

*Conclusion:* Standardization of procedures revealed that highest yield of mononuclear stem cells are obtained by in utero methods of collection, double centrifugation tube processing with cryopreservation at - 70° c.

#### Efficacy of leuco depleted red cells prepared by integral filter bags in a tertiary referral hospital

**D. Asha, P. Amalraj, R. Molly, S. C. Nair, D. Daniel**

Department of Transfusion Medicine and Immunohematology, Christian Medical College, Vellore, India

*Background:* Leucodepletion minimizes the incidence of transfusion associated adverse effects. Its major indications include the prevention of HLA alloimmunization in patients who require multiple transfusion and intrauterine transfusion. It reduces febrile non hemolytic transfusion reactions and decreases the risk of cytomegalovirus transmission. Leucodepletion can be performed either by bedside or by laboratory techniques.

*Aim:* To document the efficacy of red cells prepared by prestorage leucodepletion using Integral Sepacell filters and OPTIPRESS II.

*Materials and Methods:* Blood from donors was collected in 450 ml quadruple CPD/SAG-M blood packs with Integral 3rd generation Sepacell red cell filters. Prestorage leucodepletion was done with the help of automated blood component extractor OPTIPRESS II. Sixty leucodepleted red cell (LDRC) units were transfused to 33 patients. Among them, twelve patients were multiply transfused and none had any history of transfusion reactions. Quality assurance involved leukocyte count by manual counting using Nageotte hemocytometer and post hemoglobin checks.

*Results:* Leukocyte counting on Nageotte chamber were done for 18 LDRC units and all the units showed less than 30 leucocytes per microliter.Post Hb checks showed an increase in hemoglobin levels, of which 48% showed 1 gm% increase and 51% of patients had more than 2 gm% increase respectively. All patients who received LDRC units had no transfusion reactions after transfusion.

*Conclusion:* Leukocyte removal by red cell filtration is significantly influenced by age of the red cell concentrate.

Laboratory filtration seems to provide a more standardized leukocyte reduced product than bed side filtration and is more conducive to quality control checks. Therefore prestorage leucodepletion is essential as this prevents the release of cytokines, antigenic fragments and possibly virus or bacteria from disintegrating leucocytes. Although implementation of leucodepletion in India will lead to a rise in cost, the advantages of leucodepletion outbalance the cost.

#### Leucodepleted red cells – need of the day

**A. S. Agrawal, N. M. Bhatnagar, M. R. Mittal, J. T. Jambukiya, M. D. Gajjar, B. H. Parmar**

Dept. of Immunohematology and Blood Transfusion, Civil Hospital, Ahmedabad, India

*Background:* Blood Banking has undergone revolutionary changes in the past few decades from better testing part to switching over to components from whole blood. These days there is continuous demand for leucodepleted (LD) blood products for making transfusions safer. The main culprit behind adverse transfusion reactions (ADR) are leucocytes. For leucodepletion various filters are available in the market for both bed side and laboratory use.

*Materials and Methods:* We compared Lab side Imugard III RC-4P filter, of Terumo Penpol (TP) and Baxter inline filter.

*Results:* Leucodepletion observed was comparable in both but added advantages with TP filter is requiring less time, around 5 minutes for the procedure as compared to 45 minutes with Baxter filter. The loss of RBC was found high with Baxter filter, but there was more than 90% recovery in TP filter.

The other disadvantage with Baxter filter was the loss of costly bag in case unit comes positive during ELISA testing, bag punctured during centrifugation at the time of component separation, and the cumbersome process of priming, whereas TP filter is attached with TSCD after component separation, which gives sufficient time to test the unit. As TP filter is made of Polyurethane the hemolysis during storage is less as compared to Baxter in line filter bag made of polyster.

*Conclusion:* With our experience of more than 100 units each, TP filters having polyurethane are found to be highly bio-compatible, easy to use, cost effective, time saving and are a better option to ensure prestorage leucodepletion.

#### Assessment of T-Cell proliferative responses in blood units gamma irradiated with various doses and stored subsequently

**R. B. Sawant, N. N. Joshi, S. Hake, P. Nagaraju, A. A. Tirlotkar**

ACTREC, Tata Memorial Center, Kharghar, Navi Mumbai, India

*Background:* Transfusion associated graft versus host disease can be prevented by treating cellular blood products with gamma irradiation (GI). Variations exist in the dose and routine protocol for GI.

Aim: To assess the influence of various doses of GI and subsequent storage on T cell proliferative responses in blood units. Methods: Whole blood units (N=5 each) were GI using a Cobalt – 60 source on day-1 with 15Gy, 25Gy, 50Gy dose. Samples were collected at 1 hour, 24 hours, 72 hours, 5 days and 7 days intervals after GI and analyzed for PHA induced T- cell proliferation in the presence of IL2. Non-GI whole blood units (N=5) served as controls.

*Results:* Compared to responses prior to GI, the proliferative response of lymphocytes was 34. % with 15Gy, 18.8% with 25Gy and 1.5% with 50Gy at 1 hr post- GI. Activated T- lymphocytes and NK cells (lymphocytes responding to IL2 alone) were more sensitive to GI. By day-3, a significant (50%) decline in the proliferative response of the T- lymphocytes was observed in the whole blood units stored without GI. This response further declined to a negligible level (<10%) by day-7. A linear co-relation between % T-cell proliferative response and dose of GI (*P*<0.001) was seen at 1 hr post-GI. Thus the effect of GI on inactivation of T-lymphocytes was dose dependent. Regression analysis (R2=0.82) suggested proliferative response of 13% at 30Gy and 8% at 35Gy.

*Conclusion:* For GI of blood units more than 3 days old, a dose lower than 25Gy may suffice. However, a higher dose (35Gy) may be required for GI of fresh blood units for immediate release.

#### Does leucodepletion followed by gamma irradiation performed at various time periods during storage affect the quality of red blood cells?

**R. B. Sawant, K. M. Chawan, S. Kannan, P. Nagaraju, A. N. Marathe**

Tata Memorial Center, ACTRC, Kharghar, Navi Mumbai, India

*Background:* Leucodepletion (LD) and Gamma irradiation (GI) of red cell units affects their quality adversely. If not performed early during their storage period these interventions may lose their relevance. Currently at our blood centre LD within day 7 and GI just prior to issue of red cells is practiced.

*Aim:* To identify the safest and logistically feasible protocol for LD and subsequent GI of red cell units. Material and Methods: Six protocols were designed depending upon whether LD and / or GI were done on day 1, day 7, and day 14 of storage. Laboratory LD filters and blood irradiator with Cobalt (60) source were used for LD and GI respectively. Packed red cells (pRBC) (N=30) with SAGM or Adsol were studied. pRBC units (N= 5) which were not LD or GI served as controls. All units were stored up to day 28, sampled every week and analyzed for hemolysis, K+, LDH, pH and hematological parameters.

*Results:* Hemolysis, K+ and LDH increased with storage. LD and GI, both caused a significant increase (*P* < 0.01) in hemolysis of red cells. The protocol of LD on dayl and GI on dayl4 with further storage up to day 28 was found to be the safest protocol (R2=0.859). LD performed beyond day 7 and GI beyond day 14 was associated with unacceptable levels of hemolysis on day 28 of storage. LD showed significant (*P* < 0.05) reduction in GI induced hemolysis but had no effect on K+ leakage.

*Conclusion:* Due consideration to age of the unit and storage period after GI should be given. LD preferably on day 1 and certainly before day 7 and GI up to day 14 followed by subsequent storage for 14 days is suggested as safest and logistically feasible protocol.

#### L carnitine: Role in prevention of gamma irradiation induced red cell oxidative injury during storage

**Katharia R, Chaudhary RK, Agarwal P.**

Department of transfusion Medicine, SGPGIMS, Lucknow, India

*Background:* Transfusion associated graft versus host disease (TA-GVHD) is a devastating, usually fatal complication of blood transfusion and results from the engraftment and clonal expansion of allogenic donor leukocytes. Gamma irradiation prevents TA-GVHD but in turn damages red cells and reduces their survival. The aim of the present study was to investigate oxidative injury to red cells during gamma irradiation stored for a period 28 days, its correlation with markers of red cell membrane damage and prevention of these deleterious changes by addition of L carnitine.

*Materials and Methods:* A total of 30 red cell units included in the study were divided into two study groups. Group I were irradiated red cells without L carnitine and group II were irradiated red cells with L carnitine. These aliquots were stored at 40C for 28 days and samples were withdrawn aseptically on day 0, day 14 and day 28 of storage. Markers of membrane damage including plasma Hb, plasma K+, lactate dehydrogenase (LDH) and markers of oxidative injury such as malondialdehyde (MDA) levels, hemoglobin oxidation and osmotic fragility were studied in all samples.

*Results:* A statistically significant (*P* <0.005) increase in the mean values of plasma Hb, plasma K^+^, LDH and markers of oxidative injury such as MDA and Hb oxidation was observed in both the groups over the storage period of 28 days. L carnitine provided a protective effect against red cell membrane damage after gamma irradiation. The plasma Hb was significantly higher in irradiated red cell without L carnitine (mean plasma Hb 0.363±0.07 gm/dl) while comparing it with irradiated red cells with L carnitine (mean plasma Hb 0.281±0.06 gm/dl).

*Conclusion:* Irradiation of red blood cells can leads to oxidative injury resulting membrane damage and lysis. L carnitine provided protection against red cell membrane damage during gamma irradiation stored for a period of 28 days, which was evidenced by decreasing levels of plasma Hb, plasma K+ and LDH in L carnitine treated cells compared to those without L carnitine.

#### Comparative study of leucoreduction using different commercially available prestorage and bed side leucoreduction filters

**A. D. Sen, A. Khetrapal**

Blood Bank Armed Forces Transfusion Centre Delhi Cantt New Delhi, India

*Background:* Removal of leucocytes from cellular blood components is associated with reduction of several transfusion associated adverse reactions. Aims: To compare the efficacy of pre-storage leucoreduction vis-à-vis bedside leucoreduction using different leukocyte filters at different temperatures and assess requirement of universal leucoreduction.

*Materials and Methods:* 400 units of packed RBC were subjected to leucoreduction at room temperature and 40C using different commercially available pre-storage and bedside filters (Terumo Penpol Immugard III and Pall Medical BPF 4) and pre-filtration and post-filtration parameters were compared.

*Results:* Mean pre-filtration red cell volume ranged from 127.9 - 137.6 ml with mean hematocrit of 66.6 - 69.1% and mean leucocyte count of 8694 - 12057/μl. Mean post filtration red cell volume ranged from 118.3 - 127.9 ml with mean hematocrit of 66.7 - 69.4% and mean leucocyte count of 1.12 - 1.88/ μl. Mean post-filtration red cell recovery ranged from 88.49 - 93.49% with all bags showing more than 85% red cell recovery. Mean post-filtration residual leucocyte count ranged from 0.205 × 106 - 0.338 × 106/bag with all bags showing more than Log 3 leucoreduction.

*Conclusion:* Pre-storage leucoreduction achieved by the Terumo Immugard polyurethane filter was better than that achieved by the Pall BP4 polyester filter. Both the Terumo and Pall bedside filters showed a better performance at 4 °C. However, there was no significant difference between the two bedside filters at either temperature. Red cell recovery with the bedside filters at room temperature, their functional temperature, was significantly less than that with prestorage filters at either temperature. This study suggests that pre-storage leucoreduction is preferable over bedside leucoreduction and that polyurethane filters are better than polyester filters since the leucoreduction achieved with the former is higher. In view of the cost factor, we recommend universal leucoreduction be implemented only up to Log 1 with selective Log 3 leucoreduction for patients with specific indications.

#### Therapeutic plasma exchange in gullian barre syndrome: The experience of our center

**M. R. Mittal, N. M. Bhatnagar, A. S. Agrawal, J. T. Jambukiya, M. D. Gajjar, B. H. Parmar**

Department of Immunohematology and Blood Transfusion, Civil Hospital, Ahmedabad, India

*Background:* Therapeutic plasma exchange (TPE) has been used for the treatment of neurological diseases in which autoimmunity plays a major role. Although there are several modes of plasmapheresis applicable to GBS, no definite guidelines for selecting an optimum mode have been established so far.

*Materials and Methods:* We used Continuous flow cell separator CS-3000 for TPE. We reviewed the medical records of our patients who had consecutively been treated by therapeutic plasma exchange from March 2008 to September 2008.

*Results:* Total 36 cases were reviewed in which TPE was generally given every other day for all of the patients and one plasma volume was exchanged for each cycle. The outcome was compared and results were seen as improvement in the patient's clinical symptoms after each cycle. During the TPE procedures, none of the patients had any morbidity or mortality, and the complications were mild and manageable such as hypotension, hypocalcemia and mild anemia.

*Conclusion:* The treatment is cost effective in comparison to the available i.v. immunoglobulins. TPE is an easy and effective treatment in GBS.

#### Donor variables affecting the yield in single donor platelets

**Gupta AK, Kumari S**

Sunder Lai Jain Hospital, Delhi, India

*Background:* Emerging issues, particularly the yield in apheresis platelets and cost may affect the optimal platelet transfusion dose. Thus, we evaluated the effects of various donor factors affecting the platelets yield in Single Donor Platelet (SDP)

*Materials and Methods:* 201 SDP were prepared by two apheresis methods involving different harvest procedure, using Amicus or MCS+. Total duration of apheresis procedure and the amount of whole blood processed were noted. A total of 43.7% (n=88) SDP were evaluated for Mean Platelet Count, Mean Platelet Yield and Mean Platelet Volume (MPV) in reference to pre-donation parameters of donors.

*Results:* On an average, 2827 ml blood was processed in mean time of 67.1 min. Mean platelet count and Mean platelet yield were 1011 × 103 /ul and 2.84× 1011 respectively in SDP. Mean platelet yield was 2.42, 2.64, 2.69 and 3.19 (× 1011) for the <2000ml, 2001-2500 ml, 2501-3000 ml and >3000 ml blood processed respectively. Donor Mean Platelet Volume bears a direct relationship with platelet yield. Higher the MPV of donor more was the mean platelet yield. Donor MPV between 6-8 fl gave the optimal yield. The higher value of mean MPV in SDP was also indicative of a higher platelet yield. Mean platelet yield was 2.79, 2.82 and 3.20 (× 1011) with MPV <7.0 fl, 7.1-8.0 fl and >8.0 fl respectively in end product. Lower MPV in SDP suggests separation of young, dense platelets while higher MPV suggests separation of large platelets of better quality into SDP. Age, weight, height, hemoglobin of donor has shown no influence on platelet yield in SDP.

*Conclusion:* It is suggested that platelet yield in SDP is also affected by MPV of donor, and should also be taken into consideration while selecting a donor for optimal yield

#### Donor safety issues in high dose platelet collection using the latest apheresis systems

**R. Chaudhary, S. S. Das**

Department of Transfusion Medicine, SGPGIMS, Lucknow, India

*Background:* The practice of high yield or double dose platelet (DDP) collection through automated apheresis is gradually increasing. A low normal platelet count in our donor population makes it difficult to obtain eligible donors for such procedures. The present study highlighted the process of DDP donor selection, the product quality and safety issues during these procedures performed on donors with low normal platelet count.

*Materials and Methods:* Over a period of 14 months, a total of 67 DDP procedures were performed, 32 and 35 each on the double needle (DN) Fenwal Amicus and Fresenius(COM.TEC) separators, respectively. All procedures were performed following the departmental standard operating procedure (SOP) (Fig. 1). All results were depicted as mean ± SD and a ‘p’ value of < 0.05 was considered statistically significant.

*Results:* We observed a significantly higher platelet yield with the Amicus (*P*=0.03) and a higher volume of platelet with the Fresenius (*P*=0.00). WB processed by the Fresenius was significantly higher when compared to Amicus (*P*=0.00). Mean values of procedure related parameters such as ACD volume, DT, NT, PT and WBP and donor related parameters like citrate toxicity and vasovagal reactions were significantly higher during DDP than single dose platelet (SDP) collection (*P*< 0.05). Hb decrement after plateletpheresis was significantly higher with the Amicus (*P*=0.03).

*Conclusion:* We conclude that obtaining eligible donors for DDP from a shrinking donor population with low normal platelet values is a difficult task. So, each transfusion service should set their own guidelines for DDP collection for the interest of donor safety and optimum product quality.

#### Evaluation of nine cases of plasmapheresis: Evidence based transfusion practice at niims

**S. Bonagiri, G. R. Sastry, A. K. Meena, R. Borgohain, S. Kaul**

Department of transfusion medicine nizam's institute of medical sciences, Hyderabad, India

*Aims:* Evaluation of Plasmapheresis in nine cases, clinical condition, course of procedure and to discuss the efficacy of therapeutic plasma exchange.

*Materials and Methods:* All cases performed during August 1st 2008 to 30th September 2008 were included in the study. The age, sex, clinical condition, stage of the disease number of cycles, course of the procedure and results were noted in each case. The procedure was done with Hemonetics Apheresis Instrument by Intermittent flow cell separation method.

*Results:* Total nine cases, who under went Plasmapheresis included six males and three females. Four cases are of Myasthenia gravis. Among these three cases were associated with thymoma. In this one case is refractory myasthenia gravis post thymectomy, ten years back. Another case is sero-negative myasthenia gravis. In all the four cases plasmapheresis was done, number of cycles ranged from 1-5 depending on the condition of patient. Three cases improved significantly, clinically. One case of refractory myasthenia gravis succumbed to death; cause of the death may be citrate toxicity/ thromboembism/ cholinergic crisis. Three cases of Guillain- Barre' (GB) syndrome of Hugh's Stage IV-V were improved significantly Two cases of Chronicinflammatorydemyelinating polyneuropathy(CIDP) showed improvement clinically. Severe adverse reaction occurred only in one case. In others only citrate toxicity was seen.

*Conclusion:* Efficacy of Plasmapheresis in Myasthenia gravis and GB Syndrome stage IV - V is established. In CIDP it has to be studied more. Continuous monitoring of the procedure is required to avoid citrate toxicity. Use of fresh frozen plasma (FFP) and hypercoaugulabilty in the patients has to be evaluated further.

### Transfusion practice

#### Study of post transfusion reactions in the thalassemics using RBCS and leucodepleted RBCs

**S. S. Kadadi**

Smt. Gopabai Damani Blood Bank I.R.C.S. Solapur, India

*Background:* Any untoward event occurring in a patient following transfusion of whole blood or components is called transfusion reaction. Multiple transfused patients like thalassaemics are known to develop agglutinins. Leukocyte removals from blood components before transfusion prevent or reduce the incidence of Leukocyte mediated adverse reactions. Evidence of such study will help to provide better patient's care.

*Aim:* To evaluate post-transfusion reaction of blood in thalassaemics with RBC's. leucodepleted RBC's prepared from manual washing and using Top and Bottom Quadruple Blood Bags by Semi Automated Plasma Expresser.

*Materials and Methods:* Three different types of Red blood concentrates were used in this study. One hundred thalassaemics who received transfusion from our center are included in this study. These thalassaemics received transfusion on an average once in three weeks. Thalassaemics of ages between 5–15 years were included in this study. Red blood concentrates prepared from whole blood using double bag and triple bag. Leukodepleted R.B.C.'s are prepared by manual washing with saline and Semi Automatic method using Top and Bottom Quadruple Blood Bags.

*Result:* Post transfusion reactions with RBC's transfusion are (3.5%), with washed RBC's (1%) and with Leucodepleted RBC's prepared from Quadruple Blood Bags (0.25%).

*Conclusion:* Use of Leucodepleted RBC's reduces the incidence and severity of a number of adverse transfusion effects. Post transfusion reactions in thalassaemics are found minimum in our study using Quadruple Top and Bottom Blood Bags.

#### Platelet transfusion practice during dengue fever outbreak

**G. Kaur, S. Basu, R. Kaur, P. Kaur**

Dept of Transfusion Medicine, Government Medical College and Hospital, Chandigarh, India

*Background:* Dengue fever, dengue hemorrhagic fever and dengue shock syndrome have emerged as a public health problem in our country particularly North India. Bleeding in dengue is one of the dreaded complications and is associated with high mortality. Although the role of platelet transfusion therapy in patients with bleeding manifestations is well established, the exact indications and transfusion trigger vary greatly in different clinical settings.

*Aim:* The present study has been designed to perform a retrospective analysis of platelet transfusions during a two months period among patients with suspected dengue fever. The platelet transfusions would be analysed for the indications, pre-transfusion platelet count, platelet dose and post-transfusion increment wherever available.

*Materials and Methods:* The study included all patients who were admitted in the emergency medicine department with fever and thrombocytopenia. The clinical history (fever, myalgia and bleeding manifestations) and laboratory investigations will be analysed.

*Results:* During the two month period, 225 patients of suspected dengue having fever with thrombocytopenia received 531 units of platelet rich plasma, platelet concentrates and single donor plateletpheresis units. Dengue viral serology was done in less than half of these cases. Details of bleeding manifestations were not available on the requisition form and had to be retrieved from case files and were noted in ll%of cases (96 cases analysed so far). Platelet transfusion trigger was 20,000/μl for most of patients. Correlation of platelet count with clinical history, platelet transfusion dose and platelet count increment will be evaluated where possible.

*Conclusion:* Since large number of platelet transfusions were given with pre-transfusion count more than 20,000/μl, there is need for development and implementation of uniform guidelines for optimal utilisation of platelets. Close interaction and co-ordination between clinicians and transfusion medicine specialist is required to ensure adherence to formulated guidelines. Blood centres must have efficient inventory management and ensure prompt platelet availability.

#### Bloodless surgery (CABG) - In a patient with rare blood group

**G. N. Goudar**

Apollo Hospital, Bangalore, India

Sample received at Blood Bank from a patient aged 54 yrs, male for blood grouping and Rh type. Further 2 units of packed cells was requested for CABG the next day. On testing the blood sample, forward group was A POSITIVE, the reverse showed a strong reaction with B group cells and a weak reaction was observed with A group cells under microscope. Anti A LECTIN showed no agglutination, Anti H 3 + agglutination. This suggested the group was not A 1, on further analysis the group was A 3. The patient was posted for CABG and surgeon was informed about the rare group and also the rarity in finding the same group donors. On further discussion O group packed cells was cross matched for the surgery. The CABG with 3 grafts done off pump on beating heart. No blood was used although O group packed cells was crossmatched. Post op recovery was uneventful. On FOLLOW UP after 4 months haemoglobin was 15.2 g/dl.

#### Factors affecting platelet increment after transfusion of whole blood derived vs apheresis platelets in an oncology set up

**R. B. Sawant, A. N. Marathe, N. Khattry, A. A. Tirlotkar, S. Kannan**

Department of Transfusion Medicine, ACTREC, Tata Memorial

Center, Kharghar, Navi Mumbai, India

*Background:* The therapeutic efficacy of platelet preparations depends on various factors related to the product and patient. Aim: To analyze factors affecting platelet increment after transfusion of whole-blood derived (RDP) vs Apheresis Platelets (SDP) in cancer patients.

*Materials and Methods:* All platelet units were evaluated for Platelet count, WBC content and volume prior to issue. The CCI was evaluated at 1 hour and 18 or 24 hours. Platelet transfusions were randomized rather than patients. Multiple linear regression analysis of CCI with variables related to the patient and platelet product was performed.

*Results:* 279 transfusion episodes in 69 patients were studied. SDP's had higher (*P*=0.003) platelet content. 62% of platelet transfusions were ABO compatible. 5.7% transfusions were therapeutic and bleeding was controlled in all patients irrespective of the platelet preparation used. CCI at 1 hour and 24 hours was equal with RDP and SDP transfusion. 24 hour CCI with ABO compatible SDP transfusion was significantly greater (*P*=0.06). 29% of the platelet products were leucodepleted. Leucocyte content of RDP's was greater than SDP's. Transfusion reactions occurred in 17% patients and were associated more (*P* = 0.001) with RDP transfusions. 5.8% patients had refractoriness to platelet transfusion. The mean interval between two platelet transfusions showed no correlation with the platelet type and dose transfused. Multiple linear regression analysis revealed platelet dose, leucodepletion, shelf life of product, fever, infection, splenomegaly, bleeding, and number of transfusions received in the past as key factors affecting the CCI.

*Conclusion:* The factors affecting CCI are independent of the type of platelet preparation used. Patient related factors like fever, infection, splenomegaly, bleeding, and number of transfusions received in the past which decrease the increment after platelet transfusion should also be considered for planning an appropriate platelet transfusion support strategy in cancer patients.

#### Comparison of hemoglobin levels using hemocue hemoglobinometer with coulter counter

**S. Khan, R. N. Makroo, V. Raina, M. Chowdhry**

Transfusion Medicine Deptt, Indraprastha Apollo Hospital, New Delhi, India

*Background:* The HemoCue B-Hemoglobin analyzer is a portable, rapid and accurate method of measuring hemoglobin at the bedside. It is particularly useful in acute clinical situations and as a guide for blood transfusion requirements. It is easily used by any healthcare workers after a short period of training.

*Aims:* To compare the measurement of hemoglobin concentration [Hb] using the HemoCue haemoglobinometer with that using the Coulter haemoglobinometer

*Materials and Methods:* Two hundred (200) donors presenting to the Department of Transfusion medicine, Indraprastha Apollo Hospitals, New Delhi, were screened for hemoglobin levels using HemoCue and compared with Beckman coulter counter LH 500. A correlation was sought between the values obtained by the two methods to evaluate the following:

1. To determine the accuracy of Hemocue haemoglobinometer to measure [Hb] in our blood bank and
2. To determine the comparability of HemoCue haemoglobinometer with that of the Coulter haemoglobinometer

*Results:* Mean hemoglobin using the HemoCue is 14.49mg% (range 10-17.6mg %) and that of Beckman Coulter counter LH 500 is 14.84 (range 11.7-17.7mg %).The mean of difference in Hb levels is 0.54 (range 0-4.5). The standard deviation of the differences of the 200 samples was 0.621. The limits of agreement of the two methods (mean difference ± 2SD) were 1.78 and 0.7.

*Conclusion:* The HemoCue Hemoglobin analyzer is a portable, rapid and accurate method of measuring hemoglobin as a guide for blood donor selection.

#### Transfusion alternatives in liver transplantation: Experience from a single center

**Surekha Devi A.**

Department of Transfusion Medicine, Global Hospitals, Hyderabad, India

*Background:* Orthotopic liver transplantation (OLT) is often associated with excessive blood loss and hence, the need for transfusion of large amounts of blood products. Increased transfusion requirements are associated with sepsis, prolonged stay in the intensive care unit, higher rates of graft failure and patient mortality. Hence, transfusion-free surgery is gaining much needed attention.

*Objective:* To study the effect of intraoperative red cell salvage, antifibrinolytic drugs and recombinant factor VIIa (rFVIIa) on requirement of blood products during OLT in patients with end stage liver disease, reporting to a single center. Methods: Consecutive patients who underwent OLT at this center during the period February 2003-September 2008 were the subjects of this study. Intraoperative red cell salvage was done wherever possible. Based on the individual anesthesiologist's preference, patients were assigned to receive either 2 million units of aprotinin (AP) as a bolus followed by 500,000 units / hour or 10 mg/kg tranexamic acid (TA) as a bolus followed by 5 mg/kg every 6-8 hours, administered from the induction till the end of the surgery. When the patient had massive bleed during OLT which did not respond to antifibrinolytics and appropriate blood component therapy, rFVIIa was given in a single dose of 2.4 mg slow IV. Transfusion policy was standardized in all patients.

*Results:* 60 patients (47 M / 13 F, 52 adults, 8 pediatric patients) underwent OLT in the study period. Intraoperative red cell salvage was done in 31 patients with a median volume of 800 ml (90-4500 ml). These patients received a median number of red cells 9(1-34). 14 patients were given AP, 32 patients were given TA and 14 patients did not receive any of the agents (control group). The median volume of total blood components transfused in antifibrinolytic group (n=46) was 4670 ml (0-19,200 ml) and that of control group(n=14) was 4890 ml(0-15,500 ml). 3 patients were given rFVIIa, after which blood loss was reduced in 2 patients and received lesser blood products.

*Conclusion:* There is definite decrease in transfusion requirement during OLT in patients who received antifibrinolytics, rFVIIa and who underwent red cell salvage. Autologous transfusion of salvaged blood during surgery reduced demand on blood bank supplies. In a major surgery like OLT, which involves massive blood transfusion, use of transfusion alternatives help in conserving blood, which in turn minimizes transfusion related complications.

#### Sudden increased incidence of transfusion reactions from a ward: Root cause analysis

**A. Dubey, A. Verma, A. Sonker, S. Sachan,**

**R. K. Chaudhary**

Department of Transfusion Medicine, SGPGIMS, Lucknow, India

The transfusion medicine department of our institute was inundated with reports of transfusion reactions with red cell transfusions with patients admitted to hematology ward. Buffy coat removed red cells were routinely being transfused to these patients without frequent reaction previously.

No ABO and Rh (D) blood group discrepancy or incompatible cross matches with pre and post transfusion samples were detected. Immunohematological workup was also inconclusive. No evidence of hemolysis was found in the post transfusion blood sample of recipient. No evidence of hemolysis or clot in the blood bags was observed. Also no incompatible intravenous solution was added in the bag. The blood units were negative for bacterial culture. It was revealed that very recently a new blood administration set has been introduced in the ward. To substantiate our suspicion we allowed the blood to pass through the same transfusion set, and collected it into a transfer bag attached aseptically. The sample taken from the transfer bag showed gross hemolysis. The values of plasma Hb were 1.81 and 0.18 gm/dl at 5 min and 15 min respectively after filtration. This simple experiment identifies the probable cause of adverse transfusion reactions due to poor quality of blood administration set.

#### Prevalence and distribution of ABO and RHD antigens along with its sub-groups and rare types in greater gwalior region

**D. C. Sharma, Sunita Rai, Satya Sao, Navneet Agrawal**

Blood Bank, G.R. Medical College, and Emergency Blood Bank, Gwalior, India

*Background:* Among a total of 29 human blood group systems and over 600 different blood group antigens discovered so far, ABO and Rhesus are the most important blood group systems. The most significant rhesus antigen is Rh-D because of its immunogenicity. The sound knowledge of ABO and Rh-D antigens and its distribution in population is essential for the effective management of blood transfusion services.

*Aims and Objectives:* Study is aimed to provide data on ABO and Rh-D distribution in Gwalior region and it's comparison with related studies in India and abroad.

*Materials and Methods:* Total 90,000 blood samples were tested for complete ABO and Rh-D grouping over a period of four years by slide and tube method using complete range of anti-sera. Saliva grouping, absorption- elusion method and anti-globulin tests were performed as and when necessary. Data of frequency of ABO and Rh-D antigens were reported in simple percentage.

*Results:* In the study, the most common blood group was B (37.5%), followed by O (30.8%) and A (22.7%). The least common blood group was AB (9.0%). Among A and AB group, incidence of A2 and A2B sub-group was 8.0% and 8.6% respectively. The rare types i.e. A inter- 5, A3 -3, Ax-2, A3B-2 cases were also reported. In cord blood samples the incidence of A2 and A2B was 18.6% and 20.2% respectively. Four cases of weak-B and one case of pseudo-B were also reported. The Bombay phenotype (Oh) was not encountered during the study. The prevalence of Rh-D negative was 8.9%. Incidence of Du variant/Weaker D was 0.36%.

*Conclusion:* We conclude that most common blood group is B. The prevalence of Rh-D negative is higher than the other studies. The blood groups A2 and A2B are more frequent in cord blood / neonates than in adults

### Immunohematology

#### Comparison of gel technique with conventional tube test for indirect coombs cross match in thalassemia major patients

**J. H. Sharma, S. N. Bhagwat, S. Kawane, S. Jadhav**

Blood Bank, Department of Pathology, seth G.S.Medical College and K.E.M. Hospital, Parel, Mumbai, India

*Background:* The patients with thalassemia major, being multitransfused, get multiple alloantibodies in their sera. Hence, crossmatching by Indirect Coombs Test (ICT) is required for these patients to rule out the presence of these antibodies. Traditionally, the Coombs crossmatch is performed by tube agglutination method. Recently newer technologies for these antibodies such as gel technology have been developed. The present study was performed to evaluate and compare the results of ICT crossmatch by conventional tube method and gel technology.

*Materials and Methods:* A prospective study was performed in 100 samples of thalassemia major patients who had received more than 100 red cell transfusions in the past. ICT cross match was performed on these 100 samples using conventional tube method using pooled ’O’ cells and by gel technology using LISS Coombs cards and LISS Diluent-2 (Diamed). All the 100 samples were screened for antibodies using 3 cell panels ( Diamed). The samples showing positivity with antibody screening were tested for identification of antibody using 11 panel cells (Diamed).

*Results:* The tube technique showed positive results in 6 out of 100 samples tested. The gel card technique showed positive results in 12 samples which included the above 6 samples. These 12 samples were positive by antibody screening test using 3 cell panels, remaining 88 were negative. Thus, it is confirmed that the gel technique has detected all positive cases containing alloantibodies. The 12 cases were further subjected to 11 cell panel test, which showed presence of ‘Anti C’ in 1 sample. Remaining 11 samples were panpositive. This confirms that 12 cases detected by gel technology were true positive for alloantibodies.

*Conclusions:* Gel card technique showed sensitivity of 100% and specificity of 100%, while conventional tube test showed sensitivity of 50% and specificity of 100%. This difference is statistically significant (*P*<.05). Hence, we conclude that gel technology is superior to conventional method.

#### Determination of frequency and severity of alloimmunisation in Rh-d negative pregnant females and correlation with newborn outcome

**A. Jain, M. Thakur, R. Sharma, N. Marwah, P. Kumar, S. C. Saha**

Departments of Transfusion Medicine, Pediatrics and Obstetrics and Gynecology, PGIMER, Chandigarh, India

*Background:* Hemolytic disease of fetus and newborn (HDFN) is preventable with good antenatal care. Rh-D incompatibility is the most common cause. This study was designed: 1) to determine the frequency of alloimmunisation in Rh_D negative antenatal females 2) to compare the conventional tube technique with gel technique for alloantibody detection and titration, 3) to correlate the newborn outcome with alloantibody titre and to determine if critical titer cud be predicted with gel technique

*Materials and Methods:* The study was conducted on 223 Rh-D negative pregnant females. Blood samples were collected from antenatal women and their husbands for ABO and extended Rh typing. Samples from antenatal women were tested for presence of antibodies using Diamed 3 cell screening panel by tube IAT and gel microcolumn assay at regular intervals. Titres were also compared by both methods. Impact of alloimmunisation was correlated with newborn outcomes. Statistical analysis was performed using SPSS software version 13.0.

*Results:* 39 (17.49%) out of 223 Rh-D negative females were alloimmunised. Alloantibody specificities were anti-D (16.14%), Anti-C (0.44%), Anti-Lea (0.89%), anti – Leb (.44%). Gel test was more sensitive than the tube test in antibody detection (*P*<0.001) and showed higher titres than the corresponding tube tests. However, there was no linear correlation between tube and gel titres. 23 (15%) out of 157 newborn were DAT positive and were Rh-D positive. DAT positive newborns had higher need for exchange transfusions, higher serum bilirubin levels with longer duration of phototherapy and lower hematocrit values as compared to DAT negative newborns of alloimmunised mothers. Titre of 1 in 16, 1 in 64 and 1 in 128 correlated with newborn DAT positivity, amniocentesis, values in mid-zone and above and stillbirth respectively.

*Conclusions:* Anti-D is still the most common antibody in RH-D negative pregnant females in our population. Initial antibody screening may be done by more sensitive gel test, but the titers should be performed using the tube technique. DAT positive newborns have higher morbidity in terms of exchange transfusion and phototherapy duration than DAT negative newborns. Maternal titer by tube technique correlates with fetal/newborn intervention and outcome.

#### Cell pannel study is definitely a boon and not a burden

**P. S. Rao, S. R. Joshi, K. K. Shama**

Sahyadri Speciality Hospital Blood Bank, Pune, India

*Background:* Main objective of our study is for the detection of red cell antibodies so as to ensure that selected red cell components will survive when transfused. ABO, Rh and compatibility testing is routinely carried out but unfortunately one cannot always guarantee the normal survival of transfused cells, since even if a minute number of deleterious reaction due to serological incompatibility can still occur resulting in severe transfusion reaction and even death after transfusion.

*Materials and Methods:* 450 samples (i.e. donors + patients) were taken up for the study with Diamed gel cards and cell panel for identification of red cell antibodies on routine basis.

*Results:* All donor units were (i.e.250) subjected for antibody screening using pooled “O cells” with dia-med gel cards. Patient's sample (i.e. 200) were also subjected for antibody screening. Out of which only one patient showed presence of irregular antibodies. As per the requisition group specific unit was cross matched, which showed incompatibility hence we went for further investigations.

Three panel screening was done and +2 positivity was noted in 1st and 2^nd^ cell panel where as 3rd cell panel showed +4 positivity. Further this sample was investigated for 11 cell panel antibody study which showed + 4 positivity in cell 2,5 and 10. Chart revealed presence of anti S antibody.None of the donors showed presence of any irregular anti-bodies.

**Table:** 

**DAT and IAT was performed**	**Cross matching was done as follows**

	**Patient's Bl. Group - A_1_ Positive**
DAT - +2	A_1_ Positive unit - 4 + incompatible
	O Positive ----- - 4 + incompatible
IAT - +3	O Positive ----- - Compatible
	O Negative ----- - Compatible

*Interpretation:* The cross matching results can be interpreted as Incompatibility means presence of S antigen on the red cells of the donor. Compatible means presence of S antibody in the patient but the donor is not carrying S antigen, hence it is safe for transfusion for the said patient.

*Conclusions:* S and s phenotypes are associated with present with uncommon antibodies and diminished expressions on red cells which are usually seen as immune antibodies. These immune antibodies are reactive in antiglobulin phase at lower temperatures but occasionally reported, but still found to be carrying severe HDN and HTR. Hence such donor units must be discarded to avoid post transfusion reactions. If such units are crossmatched with any other patient it may give rise that specific antibody which will be a problem for subsequently transfusion if any. Such in-compatatibility was noted even when patient i.e thalessamia was taking transfusion for the first time. Hence we come to conclusion that totalanti-body screening is must for donors and for patients when Incompatibility is seen identification of anti-body must be done with 12 cell-panel and only compatible unit must be transfused.

#### Prevalence of sub groups A2 and A2B in delhi's blood donor population: A hospital based study

**S. Pahuja, S. Bansal, A. Toppo, M. Jain**

Lady Hardinge Medical College, New Delhi, India

*Background:* Since the discovery of ABO, blood group system, transfusion medicine has come a long way. Blood group ’A” has further subgroups, important ones being Al and A2.

*Aims:* To find prevalence of two major subgroups of A: Al /A2 and of AB: A1B/A2B in Delhi's blood donor population. Methods: Study was conducted on healthy blood donors coming to RBTC, LHMC. Out of total 7000 blood donors, 1800 donors belonged to BG A and 400 donors to AB. Subgroup A2 and A2B were detected by checking agglutination with Anti Al and anti H. Anti Al antibodies in these subgroups were detected by reverse grouping with Al cells.

*Results:* Prevalence of A2 subgroup in Delhi's donor population was found to be 4.94% while that of A2B was 6.7%. 18% of A2 and 37% of A2B had anti Al antibodies in their serum.

*Conclusions:* Presence of anti Al antibodies can cause difficulties in blood grouping and can sometimes cause transfusion reactions, if not detected. Sub grouping should be done as routine to assure safe transfusions.

#### Flow cytometric analysis for determination of ‘D’ antigen density in various Rh phenotypes of north indian blood donors

**S. K. Verma^1^, R. Gupta^2^, A. Verma^1^, R. Chaudhary^1^**

^1^ Department of Transfusion Medicine, SGPGIMS Lucknow and

^2^ Department of Laboratory Oncology, AIIMS New Delhi, India

*Background:* The Rh blood group system is the most polymorphic of the human blood groups, consisting of at least 45 independent antigens and next to ABO, is the most clinically significant blood group systems in transfusion medicine. The D antigenic sites vary in different phenotypes of Rh blood group system. The antigenic densities per RBC in different Rh phenotypes in North Indian population are reported for the first time at our institute.

*Aim:* of this study was to estimate mean channel fluorescence (MCF), which were reflective of density of D antigen, in Rh D positive, Rh D negative and weak D(Du) North Indian blood donors by using flow cytometric method.

*Materials and Methods:* A total of 76 blood donor samples including Rh D positive, Rh D negative and weak D(Du) were analyzed for the density of D antigens in different Rh phenotypes using the FITC labeled goat F (ab’)2 Antihuman IgG (H+L) antibody on FACS Calibur Flow cytometer. Result: The D antigen density was maximum in R2R2 (108.06) followed by R1R1 (56.11) in RhD positive donors, rr (4.21) followed by r”r (3.67) in RhD negative donors. Density of weak D (Du) (15.27) lies between the two.

*Conclusion:* Flow cytometry is a very good tool for demonstrating minor variation in D antigen when serological methods are inconclusive. We found that, through there was overlap in MCF among various Rh phenotypes, it is possible to discriminate between RhD positive ,RhD negative and Weak D (Du) positive samples. Further studies are required on this subject, using large sample size and weak variants of Rh system.

#### Results obtained from the use of dithiotreitol (DTT) in the complement dependent cytotoxicity crossmatch to differentiate IgM from IgG antibodies in a renal transplant setting

**M. P. Chacko, S. Ravikumar, A. Palle, D. Daniel, J. T. George**

Department of Transfusion Medicine and Immunohaematology Christian Medical College, Vellore, India

*Background:* Anti HLA antibodies demonstrated by the complement dependent cytotoxicity (CDC) crossmatch generally pose a hurdle for renal transplantation. Data shows however that Ig M antibodies, particularly when they are autoantibodies, do not lead to early graft rejection. While the usual CDC crossmatch does not differentiate IgM or IgG antibodies, the use of Dithiothreitol (DTT), which denatures Ig M antibodies, can be used in the crossmatch for this purpose.

*Aims:*

1. To find the prevalence of IgM antibodies using DTT among patients who were detected to be positive by CDC.
2. To find the incidence of post-transplant complications in patients who at the time of transplant had (only) IgM antibody as demonstrated by DTT.

*Materials and Methods:* Pretransplant screening results of patients who had a positive CDC crossmatch and consequently had a DTT crossmatch performed between April 2007 and April 2008 were retrospectively analyzed. The post transplant outcome of those in this group who underwent transplantation and whose final pre-transplant crossmatch showed positive CDC, with negative CDC/ DTT results, was examined.

*Results:* Between April 2007 and April 2008, 21 patients demonstrated a positive reaction on CDC. Using DTT, 9 of these, showed IgM autoantibody alone with no demonstrable IgG reactivity. 3 Patients showed IgM alloreactivity with no evident auto reactivity. 4 patients had IgG alloantibody alone with no evidence of IgM. 1 patient showed weak IgG alloreactivity initially, which subsided with treatment. Following this he developed weak IgM auto reactivity. 4 patients showed IgM as well as IgG reactivity, 2 of whom showed a positive auto crossmatch. At transplantation, 5 patients had IgM autoantibody, and 1 patient had IgM alloantibody. No episodes of hyperacute or acute vascular rejection were observed in this group, although 1 patient with IgM auto antibody had poor allograft function post transplant, for which a renal biopsy was performed.

*Conclusion:* The DTT crossmatch is a useful tool in the context of pretransplant screening, for differentiation of IgG from IgM antibodies. The latter should not be a contraindication for real transplantation.

#### Anti HLA antibodies – Complement dependent cytotoxicity cross match vs ELISA: A retrospective analysis of 129 patients who had undergone renal transplantation

**R. Shanthi, A. Palle, Mary. P. C. Daniel. D. George T. J.^1^**

Departments of Transfusion Medicine and ^1^lmmuno haematalogy and Nephrology, Christian Medical College, Vellore, India

*Background:* The current standard of care in terms of antibody detection in the live related renal transplant programme in India involves a Complement Dependent Cytotoxicity (CDC) cross match. More sensitive platforms have been incorporated in varied centers worldwide. However, the clinical significance of positive test results needs to be tested in each centre, to decide on the clinical applicability of the same. Our centre introduced the ELISA LAT M (One Lambda) Inc. assay in October 2007. This assay specifically detects anti HLA IgG antibodies in prospective renal recipients, but is not donor specific. Aim: To define the role of ELISA LAT M (One Lambda) Inc. screen in detecting antibodies of clinical significance pre transplant in a live related renal transplant setting.

*Materials and Methods:* The ELISA LAT M assay (One Lambda) Inc.) tests patients’ sera against pooled immobilised class I and II antigens, using enzyme conjugated anti human IgG and a colorimetric substrate for signaling. The reaction is read on the ELX800NB microplate reader. Pretransplant sera from 129 consecutive patients who underwent transplant were tested on this platform. 124 of these patients had a negative CDC crossmatch, while 9 had a positive CDC crossmatch due to an IgM antibody.

*Results:* Of the 129 patient samples on which ELISA were done, 24 showed positivity. 19 of these patients had no historical positive CDC documented, while 5 had a historical positive CDC cross match due to an IgM antibody at transplant. None of the 24 patients with a positive ELISA screen showed evidence of antibody mediated post transplant complications at 4 months of follow up.

*Conclusion:* A positive ELISA screen for antibodies does not seem to be predictive of an acute antibody mediated rejection episode. How ever, further post transplant monitoring of the same is vital to see if it plays a role in predicting later graft outcomes.

#### Hydrops fetalis associated with anti JKB -A case report

**M. Rajaiah, P. AmalRaj, M. Purna, S. C. Nair, D. Daniel**

Department of Transfusion Medicine and Immunohaematology, Christian Medical College, Vellore, India

*Background:* Red cell antibody screening in the antenatal population is vital as early detection of antibodies that can cause hemolytic disease of new born will help in appropriate intervention. We report the incidental finding of an anti- JKb antibody in the serum of an antenatal woman whose sample was received in blood bank for grouping.

*Materials and Methods:* A request for blood grouping was received from a G2P1L1 mother at 29 weeks of pregnancy. Blood grouping was done using the column agglutination technique (Biovue). As per protocol, an antibody screen using the surgiscreen 3 cell panel was also done.

*Results:* The antibody screen showed a positive reaction in two cells. Antibody identification was performed using an in house 11 cell panel which revealed an anti- JKb antibody in a titer of 1:64. The pregnancy was terminated a few weeks later as the baby was found to be hydropic.

*Conclusions:*Anti- JKb, though an uncommon antibody to cause severe hemolytic disease of new born, has been occasionally reported to cause the same. We report one such possible case. Whether the cause for hydrops was multifactorial is unclear. However future pregnancies need to be monitored closely with antibody screening, early in the course of pregnancy.

#### Current scenario of pre transfusion testing

**R. P. Singh**

Department of Transfusion Medicine, The Mission Hospital, Durgapur, India

*Background:* Traditionally pre-transfusion testing in India is doing mostly by ABO/Rh confirmation of blood unit as well as patient sample followed by indirect antiglobulin testing (IAT) cross matching. Shift from IAT cross match to antibody screening is required to maximize the detectection of clinically significant antibody which can be easily missed by traditional method.

*Materials and Methods:* Various methods are available for pre-transfusion testing, right from traditional saline tube method to semi or fully automated methods, like I.D. Gel card, solid phase red cell adherence method etc.

*Results:* In routine IAT cross matching, the chances of missing of clinically significant antibodies are quite high due to either donor red cells does not express corresponding antigen( for e.g., patient with anti-jka and donor red cells negative for jka antigen) or donor red cells having heterozygous expression of the antigen against antibodies are produced, etc.

*Conclusion:* The shift from traditional IAT cross match to antibody screening is beneficial for the patients for maximizing the detection of clinically significant antibodies and minimizing post transfusion hemolytic reactions.

#### Chance detection of anti-JSA antibody in a patient

**P. Shrivastava, A. Kumar, A. Kalra, R. Kumar**

ESI Hospital,Basai DaraPur, NewDelhi, India

*Background:*Anti-Jsa is a rare antibody which is usually IgG and results from previous sensitization via blood transfusion or pregnancy. It is associated with no to moderate delayed haemolytic transfusion reaction and mild to severe haemolytic disease of newborn. We present a case which was detected by chance to have this antibody.

*Case Report:* Blood sample was received for blood grouping and cross-matching for a 35 years old female patient suffering from chronic renal failure. There was history of one unit blood transfusion 15 days ago.

Blood group was O Positive. Antibody screen using commercial cells (Dia cell I-II-III Asia) was negative. Antibody screening test was casually repeated with pooled Ol and O2 cells prepared in-house which showed 1+ reaction with Ol and negative reaction with O2 cells in both gel column agglutination as well as conventional tube (liss /AHG) test. Direct antiglobulin test and auto-control were negative. 11 cell commercial panel from Diamed was put up for antibody identification. One cell showed positivel+ reaction and rest of the ten cells were negative. This reaction pattern matched with Anti-Jsa antibody. Testing two other cells positive for Jsa antigen to satisfy 3×3 rule and phenotyping of patient's RBCs for Jsa antigen could not be performed due to non-availability of necessary cells and antiserum. The records of earlier transfusion revealed that antibody screen was negative that time and no adverse reaction was noted. O positive donor unit compatible with patient in liss/ AHG phase was issued with instructions to transfuse slowly under strict supervision. Blood transfusion was uneventful.

*Discussion and Conclusion:* Jsa is a low frequency antigen Frequency of Phenotype Js (a-b+) in white population is 100% and 80% in black population. It is not required that screening or panel cells should have Jsa + cells. It is also noted that most of the commercial antibody identification panels do not have a single Jsa + cell. All the three commercial screening cells were negative for Jsa Antigen and so antibody was missed. Pre-transfusion antibody screening is a key process to detect clinically significant unexpected antibodies. Frequency data for various antigens are mainly available for Caucasian population. The incidence may be different in Asian / Indian population. There is need to develop and market antibody screening and panel cells based on the frequency of antigens in Indian population

#### Transfusion of non-group specific platelets: Does antibody titration help?

**R. B. Sawant, A. A. Tirlotkar, A. N. Marathe**

Dept of Transfusion Medicine ACTREC, Tata Memorial Center, Kharghar, Navi Mumbai, India

*Background:* Transfusion of ABO incompatible platelets containing large volume of plasma or having high antibody titers can lead to complication like acute hemolysis in recipients. Policy regarding transfusion of ABO incompatible platelets is lacking currently in our country.

*Aim:* Screening the samples from platelet donors for anti-A and/or anti-B titers and study the outcome of transfusion of incompatible platelets in cancer patients.

*Materials and Methods:* Serum samples from platelet donors were tested for hemolysins, IgM titer and IgG titer. Standard procedures as stated in AABB Technical Manual, 14th edition were followed. Platelet transfusion outcome was measured using Corrected Count Increment (CCI) in plasma reduced ABO incompatible transfusions versus that in ABO incompatible transfusions (N = 30 in each group). IgM titer >128 and IgG titer>256 was considered as high titer.

*Results:* High IgG antibody titers were present in 16% of A group, 17% of B group and 23% of O group donors whereas high titer IgM antibodies were present in 17% of A group, 24% of B group and 27% of O group donors. 21% of anti-A and 17% of anti-B antibodies were hemolysins.31% of group O donors had high titre of both IgG and IgM antibodies. The CCI was equivalent (P=0.765) with transfusion of O group Plasma Reduced (PR) single donor platelets (SDP's) and ABO incompatible SDP's with low antibody titre transfusions in patients with matched clinical conditions. No severe adverse events were reported in both groups.

*Conclusion:* High titre ABO antibodies and hemolysins are present in a high proportion of donors (especially group O donors). Antibody titration reduces the need of further interventions like PR prior transfusion of SDP's across the ABO barrier. Further study regarding clinical outcome of transfusion of non PR ABO incompatible SDP's with low antibody titre is needed.

#### Anti-D and anti- C in prenatal serology: A case report

**D. Sachan, A. Sonkar, A. Verma, R. K. Chaudhary**

Department of Transfusion Medicine, SGPGIMS, Lucknow, India

*Background:* Pregnancy presents special immunohematological problems for the transfusion service by exhibit alloimmunization to fetal antigens. The antigen that most frequently induces immunization is D which is usually present alone and very less often, in combination with Anti-C antibodies. Differentiation of Anti -D, -C, and anti-G is necessary to determine the need for Rh immune globulin prophylaxis.

*Case Report:* A 25 year-old female, G3P2+0 was referred to our institute as a suspected case of Rh isoimmunization at gestation age of 28 weeks. Previous two deliveries were done in hospital setting and prophylactic Anti-D immunoglobulin was given as per their protocol. Patient's blood group was B Rh (D) negative. The Indirect Antiglobulin Test (IAT) was positive. To identify the red cell alloantibody further IAT was done using Diamed ID-panel. As per the antigram given in the product insert, the suspected red cell antibodies were Anti-D and anti-C. Phenotype of the patient and her husband was done Patient Rh phenotype was rr and the husband's phenotype is R1R2(D+, C+, E+, c+, e+). Individual titration was done of Anti-D and anti-C using R2R2 and r'r cells respectively. The titer of anti-D and anti-C was 16 and 8 respectively. To rule out presence of anti-G, the PEG adsorption of mother's serum was done with r'r cells as per our laboratory SOP. The adsorbed serum was tested with r'r cells by IAT along with unabsorbed serum as a control. After one step adsorption, IAT became negative with r'r cells but positive with R2R2 cells. It was thus confirmed that mother's serum contained Anti-D and -C and also ruled out possibilities of Anti-G.

*Conclusion:* Antibodies against G appear superficially to be anti-C+D but it can not be separated into anti-D and anti-C. In this case, we have separated anti-D and anti-C by differential PEG adsorption method confirming the absence of anti-G.

#### Haemolytic disease of newborn due to anti m antibody - A case report

**P. Amal Raj, R. Molly, M. Purna, Nair, S.C., D. Daniel**

Department of Transfusion Medicine and Immunohaematology, Christian Medical College, Vellore, India

*Background:* Hemolytic disease of the new born (HDN) is usually caused by Rho (D) and ABO incompatibility and is rarely associated with other blood group antigens like Kell and Duffy blood groups. The MNSs blood group is one of the earlier red cell antigen systems to be recognized and anti M is the most frequently encountered antibody in this blood group system. The prevalence of anti M immunization may be increasing but the incidence of hemolytic disease due to anti M is extremely low.

*Methods and Results:* Blood samples of a 28 year old G3A2L0 mother and her 8 day old baby were sent to our blood bank for blood grouping and antibody identification. The baby had been delivered at about 35 weeks gestation after hydrops fetalis was detected on an ultrasound scan. The baby's Hb at birth was 4.4gm%. Blood grouping done using the column agglutination technique showed the mother's blood group to be O Positive and the baby's B Positive. The Indirect Coombs Test was positive with a titre of 1:128 and direct coombs test was negative on the mother's blood sample. Direct Coombs Test of the baby was negative. Antibody identification with an in-house cell panel identified anti -M antibody in the mother. Phenotyping showed the mother to be negative for M antigen and the father and baby to be positive for the M antigen.

*Conclusion:* We report this finding as a case of fetal hydrops with hemolytic disease due to anti -M isoimmunization in the newborn. Literature review shows that, though the majority of anti- M antibodies are IgM, an IgG component frequently co-exists which may cause Hemolytic Disease of Newborn of varying severity. It is therefore vital that all antenatal patients be screened for the presence of red cell alloantibodies, so that appropriate clinical interventions can be made.

#### Presence of anti-M antibody in normal healthy donor without history of blood transfusion and pregnancy

**L. Jagannathan, A. Mathur, Joyce**

Rotary TTK Blood Bank, Bangalore Medical Services Trust, Bangalore, India

*Background:* The prevalence of red blood cell (RBC) alloantibodies among general, hospital-based patients typically has averaged approximately 1 percent in various studies. They are alloimmunized as a result of transfusion or pregnancy. The presence of irregular antibodies in healthy voluntary blood donor is uncommon. In donors also alloimmunization is due to previous transfusion or pregnancy. But rarely can it be found in sera of persons who have had no exposure of human red cells. Sometimes these can be clinically significant and become problem in immunohematology lab. We present such a case found in our blood bank.

*Case Report:* The donor ID A22087 donated blood voluntarily on 12^th^ August 2008. Cell grouping was showing “A” Positive but in serum grouping positive reaction (+3) was seen with all the three reagent red cells A-cell, B-cell and O-cell. The tests were repeated on the ID-Card, DiaClon ABO/D + reverse grouping (Gel Card). Same results were observed with gel cards. When reverse tubes were put in three different temperature 4C, 22C and 37C for one hour, reaction remained same with 22C and 37C but negative with 4C tube. The sample was reactive with anti-Al lectin and H lectin. Autocontrol and DAT were negative. Finally unexpected antibody was suspected but when donor history was seen, he was a male donor without any history of transfusion, so the presence of an antibody was considered unlikely.

With antibody screening cell panel, two out of three cells were showing positive result and with the antibody identification panel (ID-DiaPanel 1-11 by DiaMed), suspected antibody was anti-M. The most likely type of antibody was IgG as the plasma was reactive even after treating with Dithiothreitol (DTT). DTT destroys the IgM antibodies.

The A1 and O cells used for reverse grouping were shown to be positive for the M antigen. The anti-M present in the sample was interfering in reverse groping with A1 and O cells. The A1 and O cells treated with papain enzyme gave a negative reaction in reverse grouping, as papain destroys the M antigen. This further confirmed the presence of and Anti-M with a wide thermal range reacting at room temperature as well as 37C, therefore clinically significant.

*Conclusion:* Anti-M is often found in sera of persons who have had exposure to human red cells. Many examples of Anti M are cold reactive, saline agglutinating, non red cell stimulated antibodies, predominantly of the IgM type. However, this was a rare case of a non red cell stimulated IgG Anti M which can be clinically significant as well. Therefore antibody screening is recommended in all donor samples.

#### Red cell alloimmunization in multi-transfused patients

**S. Sachdeva, P. Kaur, S. Basu, R. Kaur, G. Kaur**

Department of Transfusion Medicine, Govt. Medical College and Hospital, Chandigarh, India

*Background:* Red cell alloimmunization resulting from prior exposure to foreign antigens due to transfusion is a significant complication in multi-transfused patients. Tissue transplantation or pregnancy are other mechanisms of alloimmunization. The frequency of red cell alloimmunization has been reported between 0.3 to 38% in hospital patients. Patients of thalassemia, sickle cell anaemia, auto-immune haemolytic anaemia, hemato-oncologic disorders and chronic renal failure on dialysis therapy may develop allo-antibodies due to repeated transfusions. These allo-antibodies not only interfere with pre transfusion compatibility testing but detection of these antibodies is necessary as they may adversely affect the response to transfusion, especially if they are clinically significant. It is also interesting to know whether these antibodies can be detected by in-house screening cell panel.

Aim:

1. To determine the frequency of alloimmunization in transfusion dependent patients including multiparous females compared to other patient groups with no prior history of transfusion or pregnancy.
2. To compare the results of red cell screening for allo-antibodies with commercial and in house red cell panel.

*Materials and Methods:* The study population included 100 patients. Among these 50 patients each were selected with or without history of prior transfusion or pregnancy respectively. Patients with two or more prior transfusions were included in the former category. All patient sera were subjected to antibody screening by commercial red cell panel and 'O' pooled cells prepared in house.

*Results:* The results of antibody screening obtained with commercial screening cells and ‘O’ pooled cells will be compared. The significance of employing routine antibody screening for all patients versus only for transfusion dependent patients along with its clinical implications will be discussed. The feasibility of using an in house red cell panel in place of commercial panel will be considered. So far out of 29 patients analysed with history of prior transfusion or pregnancy, the incidence of red cell allo-antibodies was 3.44%A positive correlation of alloimmunization was observed with increase in number of transfusions.

*Conclusion:* High incidence of alloimmunization in multi-transfused patients warrants the use of antibody screening and identification as a part of pre-transfusion compatibility testing. The antibody screen may however not detect antibodies to low frequency antigens not present on any of the screening cells. The study would also include the utility of an in house red cell panel at our blood centre.

#### Importance of conventional tube testing in resolving ABO discrepancy due to cold agglutinin disease

**N. Agarwal, D. Sachan, R. K. Chaudhary**

Department of Transfusion Medicine SGPGIMS, Lucknow, India

*Background:* Peripheral blood centers in India are still widely practicing only forward ABO grouping by slide method before release of blood. Certain disease conditions causing ABO discrepancies (e.g cold agglutinin disease) are missed if reverse grouping is omitted. We are presenting here a case of cold agglutinin disease, in which a patient was grouped incorrectly and mismatched transfusion was given in an outside hospital.

*Case Report:* A 18 year-old female was referred to our institute with a provisional diagnosis of hemolytic anemia and a history of transfusion with AB +ve blood 5 days before with hemoglobin 3.5gm%, reticulocyte count 15, total bilirubin 2.9 gm/dl and serum LDH 1200 IU/L.

A request was sent to our department for 2 units of PRBC. A discrepancy was found between forward grouping (O positive) and reverse blood grouping (group AB). We tried to resolve this discrepancy by repeating cell grouping after washing the sample 3 times and serum grouping after incubation at 4o C, 22o C and 37o C and the blood group was confirmed to be O Rh (D) positive.

As the patient had been transfused with AB positive blood, and also to find the cause of grouping discrepancy, we performed the detailed immunohematological work-up including Direct Coomb's Test (DCT), Indirect Coomb's test (ICT) at different phases. Patient was confirmed to be a case of Cold Agglutinin Disease with a titer of 512 with co-existing hemolysis due to mismatched transfusion.

*Conclusion:* This case highlights the importance of forward and reverse grouping using conventional tube technique to resolve blood grouping discrepancies and thus avoiding mismatched blood transfusion.

#### Prevalence and relative frequency of red cell alloantibodies in Rh negative pregnant women in an Indian population by gel centrifugation technique

**A. Khetrapal, A. D. Sen**

Blood Bank Armed Forces Transfusion Centre Delhi Cantt New Delhi, India

*Background:* Irregular antibodies in maternal serum may cause hemolytic disease of the newborn (HDN) as well as post transfusion complications if appropriate cross-match test is omitted. Gel centrifugation techniques have increased sensitivity and specificity for red cell antibody detection.

*Aims:* To determine prevalence and relative frequency of RBC alloantibodies in Rh-negative antenatal cases in India using gel technique. To evaluate whether irregular antibody screening should be adopted as a routine antenatal test for Rh-negative antenatal cases.

*Materials and Methods:* Sera from 4196 antenatal cases were investigated for Rh status between January-December 2007. The Rh negative cases were further evaluated for RBC alloantibodies by DAT, IAT and gel agglutination using a 3 cell screening panel and an 11 cell identification panel (DiaMed ID Diacell I-II-III Asia and DiaMed ID DiaPanel).

*Results:* 340 cases were found to be Rh D negative (8.10%). All cases were DAT negative. 10 of the 340 samples (2.94%) were IAT positive. Anti-D antibody was the lone antibody in 4 cases and in association with other antibodies in 4 cases (2 with anti C, 1 with anti K and 1 with anti K and anti M) showing an overall positivity in 8 cases (2.35%). Anti C was the lone antibody in 1 case and Anti K in 1 case. A total of 15 antibodies were detected in the 10 cases. Anti D represented 53.3% of antibodies detected, anti C and anti K 20% each and anti M 6.6%.

*Conclusions:* 2.35% of Rh negative cases developed immune anti-D. This high incidence of immune anti-D is probably due to inadequate protocols for Rh IG prophylaxis in India. Overall prevalence of irregular antibodies was 2.94% in Rh negative antenatal cases and 0.24% in all antenatal cases. In view of this, we recommend that irregular antibody screening by gel technique should be adopted as a routine antenatal test, especially for Rh negative antenatal cases in India, in order to improve outcome of both the neonate and the mother.

#### Prevalence of weak D in the blood donors at Blood Transfusion Centre Indira Gandhi Medical College, Shimla

**S. Malhotra, M. L. Kaushal**

Dept of Immunohematology and Blood Transfusion, Indira Gandhi Medical College, Shimla, India

*Background:* Rh blood group system is an important and complex blood group system of the human red blood cells. Antigens of this blood group system are highly immunogenic and clinically significant. An individual is designated as Rh Positive and Rh negative depending upon the presence or absence of D antigen. Weak D RBCs are historically defined as having reduced D antigen level that requires IAT for detection. There is reduced number of antigen sites on the RBCs. To prevent the unwanted immunization of D Negative individuals because of transfusions with weaker variants of D the donor blood should be screened for the presence of weaker variant of D antigen.

**Table:** 

**Immediate Spin**		**IAT**	

**Sample**	**Monoclonal IgM**	**Blend IgM and IgG**	**Blend IgG and IgM**
1.	w	1+	2+
2.	-	1+	2+

*Materials and Methods:* The study was conducted on 4850 samples collected from voluntary and replacement donors over a period of 9 months from Jan. 2008 to Sept.2008. D Antigen testing by conventional tube method was performed using IgM Monoclonal and Blend of IgM and IgG anti sera from two different manufacturers. RBCs found to be D Negative or weakly D Positive (< 2 +) were further tested for weak D by IAT. A positive result in the IAT defined weak D in this study.

*Results:* Two donors (0.04%) were classified as weak D by IgM monoclonal and Blend of IgG and IgM as shown in table. Monoclonal blend Anti D showed the stronger reaction on direct agglutination. Both the sample showed positive result ( 2+) in IAT.

*Conclusion:* The prevalence of weak D in the blood donor population comes out to be 0.04% in the present study. The production of Anti-D in D negative patients transfused with weak D has been reported. The weak D red cells should be considered as D + for transfusion. Since the molecular tests to distinguish various weak D types are not available in most of the institutions, the Rh D negative patients especially women of child bearing age and young males should not be transfused with weak D+ blood.

#### Distribution of ABO and Rh blood groups among the blood donors in a tertiary care centre of Himachal Pradesh

**M. L. Kaushal, S. Malhotra**

Department of Immunohaematology and Blood Transfusion, Indira Gandhi Medical College, Shimla, India

*Background:* The data on the distribution of blood groups in the blood donor population of Himachal Pradesh is limited as no such studies have been conducted. The present study was planned to find out the distribution of ABO and Rh blood groups among the blood donor population of Himachal Pradesh. Being tertiary care centre, the blood donation camps are being organized through out the state and hence the data represents the entire blood donor population of the state.

*Materials and Methods:* The study was conducted in the Blood Bank of Indira Gandhi Medical College and Hospital which is a tertiary care centre of Himachal Pradesh. The distribution of ABO and Rh blood groups was studied among 8633 blood donors over a period of more than one year from Jan.2007 to May 2008. The study included voluntary blood donors (VNRBD) coming voluntarily to the blood bank or in the voluntary blood donation camps as well as the replacement donors coming to the blood bank.

**Table:** ABO Blood Group Distribution

**Total No.**	**Group A**	**GroupB**	**Group O**	**Group AB**
8633	2525	2882	1945	1381

**Table:** Rh (D) Blood Group Distribution

**Total No.**	**Rh ( D)+**	**Rh (D)+**
8633	8108	525

*Results:* The most common blood group was found to be group B (2882; 33.38%) followed by group A (2525; 29.24%) and group O (1945; 22.52%).The least common blood group detected was AB (1381; 15.99%). No Bombay phenotype was detected. The prevalence of Rh (D) Positive group was 93.91 % (8108) and Rh D Negative 6.09% (525).

*Conclusion:* The commonest blood group detected in the study was group B which is similar to the data of northern region. It is closely followed by group A as opposed to group O which is the next commonest group in the data of northern region. The group O comes out to be the third commonest group followed by AB group which is the least common group. The prevalence of Rh D positive and Rh D negative group was 93.91% and 6.09% respectively which is similar to the earlier data.

#### How an immunohaematology automate would have helped!!

**S. Pathak, M. Chandrshekher, G. Wankhede**

Transfusion Medicine, Max Heart and Vascular Institute, New Delhi, India

*Background:* Despite advances in laboratory automation, a considerable amount of work in Blood Banks is still done using manual methods. However, with proper validation, techniques, and procedures, all routine blood-bank tasks can subsequently be automated. Most of the technologies used for immunohematology procedures in Blood Banks offer a range of instruments to automate and reduce errors, document and speed up processes.

*Case:* We present a case, 57 year old female patient admitted with history of bilateral osteoarthritis in the knees and mild anemia. She was admitted with a plan of Bilateral total knee replacement. She had a history of hysterectomy about 18 years back along with history of blood transfusion. Her blood group was B Positive. 4 units of Packed Red Cells were requisitioned by the clinicians. The 3 cell screen was positive with 2 of the cells. The night technician was not trained for the Antibody identification process. The same technician set up 30 crossmatches with B positive units; however, he was unable to get compatible units. The patient's antibody identification panel was also positive with no reaction in the enzyme phase and a diminished reaction at room temperature. The antibody was identified to be Anti M. We had to transfuse M negative Coombs compatible units to this patient. We screened 111 units of group 'O and 'B' Packed Red Cells (PRBC) for the presence of M antigen using the DiaMed ID-MTS single antigen cards. Out of 111 units of PRBC, we got only 6 units of M Negative PRBC, which were compatible for this patient. The time spent to carry out this whole exercise was close to 8 hours. The operation of the patient had to be postponed to another day.

*Discussion:* This is a case which convinced us to go for an automation of the immunohaematology section of the Blood Bank as well. All the patients scheduled for the surgery can undergo an Antibody screen as a protocol. An automate can screen all the samples together as a batch. The antibody identification panel and the interpretation can be done in the automate using an antibody identification software such as the VIP from DiaMed. The antigen profile could be run simultaneously. More importantly, multiple units of Blood can be tested for the absence of a particular antigen (M in this case). The hands on time could be reduced and the Antigen Negative blood can be arranged at a short notice even by staff that is not well versed with the antibody identification and interpretation process

*Conclusion:* Every Blood Bank should analyze its work load, its work pattern, ability of technicians and profile of tests it is carrying out before deciding to go for the level and type of automation in Immunohematology. Automation is the way forward for maximum security, speed, reliability, full traceability and most importantly safety of the patients.

#### Three cases of delayed hemolytic transfusion reactions

**P. Shrivastava**

ESI Hospital, Basai Dara Pur, NewDelhi, India

*Background:* Delayed Haemolytic transfusion reaction is defined as accelerated destruction of transfused red cells usually associated with an anamnestic antibody response to alloantigen(s) on donor red cells. These reactions may not be recognized for days or weeks after transfusion and often may go unreported. We present three such cases.

*Case 1:* 65 Years female, a case of severe anaemia, Blood group 0+, antibody screen negative with pooled O cells, O+ donor unit compatible in tube saline/ AHG phase was issued. Blood transfusion was uneventful. After twenty days, one more unit was requested. Antibody screen was positive this time( 4+ both Ol and O2), DAT was negative, major crossmatch was incompatible. Antibody Identification panel was not available. Sample was referred to a higher centre. Antibody identified as probably anti-C + Anti-e, not a single compatible unit could be found.

*Case 2:* 38 years male, a case of ulcerative colitis with bleeding per rectum, H/o 3 units blood transfusion one year ago. Blood Group B Pos, Antibody screen: 2+ / 0/ 0 respectively in O1,O2,O3 cells (commercial, Diamed), using gel column agglutination technique; Reactions in eleven cell antibody identification panel matched with Anti-K. Patient was negative for K antigen. Cross match compatible with B+, K negative donor unit in tube/ AHG as well as gel technique was kept ready but clinician chose not to transfuse. Consultation issued that this patient must always be transfused with K negative compatible units in future

*Case 3:* 21 year old unmarried female patient, a case of Chronic Renal failure, history of three units blood transfusion 8 months ago, blood group O Positive, Antibody screen positive, both DAT and autocontrol 2+ (mixed field), antibody identification panel pan positive. Antibody identification panel using an eluate from patient's RBCs indicated presence of anti-c and anti-E. Patient was found negative for c and E antigen. Cross match with c negative E negative O positive unit was compatible and transfused. Consultation was issued for transfusing c negative E negative O positive in future.

*Conclusion:* We identified these cases during routine antibody screening. They could have gone un-noticed if these tests were not done as a routine. There is general lack of awareness about antibody screening, identifications and implications of positive results. Most of our blood banks are not tuned to solve the problems arising due to presence of antibodies.

#### Transfusion support in a thalassemic child with anti-S alloantibody

**A. Singh, D. Sachan, A. Solanki, A. Sonker, R. K. Chaudhary**

Department of Transfusion Medicine, SGPGIMS, Lucknow, India

*Background:* Multiple transfusions in thalassemia major patients expose them to various complications of blood transfusion including Alloimmunization against red cell antigens. Once alloimmunized, transfusion support becomes more challenging in such patients.

*Aim:* To provide appropriate transfusion support to red cell alloimmunized multi transfused thalassemia patient.

*Result:* We describe the case of a 5-year old child, a patient of thalassemia with massive splenomegaly with hypersplenism. There was history of transfusion reactions at various episodes of blood transfusion. Patient was admitted for splenectomy with laboratory parameters of hypersplenism including Hb 3.8 gm/dl, platelet count 67,000/μl. Pre-transfusion antibody screen was positive which was confirmed to be anti-S antibody. Patient was transfused with 2 units of “S” negative packed red cells without any complications.

*Conclusion:* The development of anti-RBC antibodies can significantly complicate transfusion therapy in multi transfused thalassemics. Some alloantibodies are hemolytic and may cause hemolytic transfusion reactions and limit the availability of further safe transfusion. Knowledge of allo-antibodies is very important as it aids in the selection of appropriate blood suitable for transfusion to these patients.

#### A prospective study for detection and identification of red cell alloantibodies in multiply transfused thalassemia major patients

**R. Jain^1^, J. Perkins^2^, V. Harimoorthy^1^, S. T. Johnson^3^, P. Desai^1^, U. Chudgar^1^, S. Tulsiani^1^, N. Choudhury^1^**

Prathama Blood Centre, Ahmedabad, ENH Blood centre, Chicago, USA., Blood centre of Wisconsin, Milwaukee, USA.

*Background:* Life long red blood transfusion remains the main treatment for β thalassemia major patients. The development of alloantibodies complicates transfusion therapy in thalassemia patients. Alloimmunisation to red cell antigens is one of the most important immunological transfusion reaction and causes delayed type of transfusion reaction.

*Materials and Methods:* A prospective study was conducted from January 2007 to November 2008. Seventy patients were included in this study and 3 consecutive samples collected after every 6 month and investigated for the development of alloantibody to red cell antigens. Antibody screening was carried out on serum employing commercial three-cell panel using standardized blood bank techniques. If patients were found to have an irregular red cell alloantibody then the antibody identification was performed using commercial 11 cell panel cells.

*Results:* A total of 70 patients were included in the study. 52 patients were males and 18 females. At the beginning of study, the age ranged from 9 month to 22 (mean age was 9.42) years. Irregular red cell alloantibodies were found in 3 patients. Out of 3,2 patients developed antibody in 2^nd^ and 3^rd^ samples. The 3^rd^ patient showed pre existing antibody before inclusion in the study. The age of 3 patients who developed red cell alloantibody are 8, 4.2, and 12.7 years, respectively. Blood transfusion ranged from 5 to 600 units.

*Conclusion:* Red cell alloimmunisation should be kept in mind in the patients receiving multiple transfusions. In present study, alloimmunisation rate was 4.28%. Mean transfusion duration in these patients was 18 days, probably due to presence of alloantibody. RBC alloantibody detection on regular interval and antibody negative blood transfusion is strongly recommended in transfusion dependent thalassemia patients.

### Administrative

#### An audit of blood components in a tertiary care hospital of Gujrat

**M. R. Mittal, N. M. Bhatnagar, M. D. Gajjar, B. H. Parmar**

Department of Immunohematology and Blood Transfusion, Civil Hospital, Ahmedabad, India

*Background:* As there are no alternatives to human blood, judicious use of blood by using components is the only means by which the increasing demand and short supply can be met with. Different components of blood are recommended by experts for different clinical indications but whole blood is still widely used.

*Aims:* To know the utilization of different blood components in Civil hospital Ahmedabad, a retrospective audit was conducted at Department of IHBT, Civil Hospital, Ahmedabad.

*Materials and Methods:* A retrospective study of the blood bank records and electronic records of the patients who were given different blood components was done from January 2008 to June 2008. Estimated production of blood components, number of patients transfused, and number of transfusion episodes and indication for transfusion were assessed.

*Results:* Total prepared blood components were 17816 in numbers, 5.42% was the wastage rate of different components. 16591 units were transfused to 10036 males and 6555 females of different age groups. Though more number of whole blood units were issued to the cardiology department, the orthopedics department was the one with maximum usage of whole blood in comparison to the other departments (z=31.348) and pediatrics department was the department which used least whole blood. *Conclusions:* Regular audit of blood and blood components is must so that necessary measures can be taken for appropriate use. Continuous educational programmes will be helpful to decrease the irrational use of blood.

#### Transfusion audit of surgery cases: Increased awareness about component utilization

**S. C. Gupte**

Surat Raktadan Kendra and Research Centre, Surat, India

*Background:* Surat Raktadan Kendra and Research centre is the stand alone blood bank and Regional blood transfusion centre which supplies blood to > 500 hospitals and nursing homes in Surat district. Transfusion audit was carried out on five years’ data of patients undergoing orthopedic, obstetric / gynec, cancer and general surgeries to assess the trend of component utilization.

*Materials and Methods:* Patient's details provided by the clinician were entered in the Microsoft Excel. All entries were double checked and edited. If more than one requisition form was received for a patient then the supplied units were pooled together.

*Results:* Form April 2003 to March 2008,15759 patients undergoing different types of surgeries were analyzed. They received total 37142 units of whole blood, red cell concentrate, fresh frozen plasma (FFP), platelets and cryoprecipitate. The whole blood utilization reduced significantly during last five years as shown below: The maximum awareness regarding blood component use was among the cancer surgeons. In 2003 – 2004 nobody asked for cryoprecipitate but from 2006 onwards there was demand for this component also. There was no significant change in component ordered per case. On average orthopedic surgeries used 2.14, Gynecologist 2.07, cancer surgeons 3.7 and general surgeons 2.4 units/case. The single unit transfusions also gradually reduced over the years. *Conclusion:* During five years significant increase was observed in utilization of blood components for various surgeries. Whole blood and single unit demands significantly reduced and RCC, Platelets, FFP were used more frequently. This study will help to change the attitude of clinicians about blood component use for surgery and improve judicious use of blood.

**Table:** 

Surgeries	% of Whole blood used in the year		*P* Value

	2003 – 04	2004 – 05	
Orthopedic	76	55	<0.001
Obstetric / Gynec	76.5	50.6	<0.001
Cancer	38.3	31.9	<0.05
General	79.3	35.4	<0.001

#### Transfusion audit as a tool to improve clinical transfusion practices for quality management in BTS

**A. Jhaveri, M. A. Kahar**

IRCS Blood Bank, Navsari, India

*Background:* An urgent need is always felt for implementation of transfusion audit as a tool for quality management in BTS especially to improve clinical transfusion practices. When we look back and view the data for last ten years retrospectively, the scenario is disappointing, frustrating and irritating. If radical steps are not taken, it seems that we will never be able to cater the need of our country in time and in an appropriate manner.

*Aims:* To ascertain whether implementing Transfusion Audits can reduce the unwarranted transfusions and rationalize the use of scarce resource like human blood.

*Materials and Methods:* Retrospective analysis of the data of last ten years at Blood Bank managed by IRCS, Navsari as regards to types of demands received, quantity of units demanded and actually transfused, units de-reserved etc. We also reviewed the relevant literature in this context. Results: will be discussed in the presentation.

*Conclusion:* Dialogue, consultation and at times confrontation are needed with clinicians in cases of irrational prescriptions. Some legislative involvement is also needed from government agencies to curb misuse of blood. Transfusion Audits must be made mandatory by FDCA and/or Blood Transfusion Councils. ISBTI can play a key role in developing guidelines and get involved in committees etc. Active participation of end-users in the blood bank activities and functioning is want of time. Hospital transfusion committees need to be set up and regular audits are to be conducted and appropriate prescribing guidelines must be formed and necessary corrections made in mutual dialogue after review of outcome. Such hospital transfusion committees must be active and just not on paper as is the current scenario. If the indications for transfusion cannot be medically defended and the patient is injured, the physician MUST be held liable. The blood bank cannot review every decision to transfuse but may help prevent the inappropriate use of blood by regular audit of transfusions and by helping to educate the clinicians. Initially retrospective audits are needed followed by prospective and concurrent audits as soon as they are practical and possible.

#### Assessment of FFP utilization in tertiary care hospital of Gujrat

**M. R. Mittal, N. M. Bhatnagar, A. S. Agrawal, J. T. Jambukiya, M. D. Gajjar, B. H. Parmar**

Department of Immunohematology and Blood Transfusion, Civil Hospital, Ahmedabad, India

*Background:* There are no written guidelines on the use of FFP in India and there is a lot of wastage of this blood product, hence this study was undertaken. This study was conducted in the Department of Immunohematology and Blood transfusion Civil Hospital, Ahmedabad, for a period of 6 months, with the aim of making recommendations to reduce inappropriate use of FFP in the hospital.

*Materials and Methods:* A retrospective study of blood bank records and electronic records of the patients who were given FFP was done from January 2008 to June 2008. The standards taken as reference were British Committee for Standards in Hematology, American Association of Blood Banks and World Health Organization guidelines.

*Results:* 2242 units of FFP were used during the study in 1050 transfusion episodes to 788 patients for which the inappropriate requests were 968. Inappropriate requests were 43.18%. FFP was used appropriately in 56.83% cases. The department of Medicine and plastic surgery were the departments with maximum number of inappropriate requests.

*Conclusion:* In absence of any national guidelines on the usage of FFP, every transfusion centre should formulate their own guidelines according to the hospital usage. A continual system of staff education and administrative intervention will be helpful to reduce inappropriate use in future.

#### Analysis of transfusion related nonhemolytic reaction as reported by clinicians

**G. K. Patidar, A. Jain, R. R. Sharma, N. Marwah**

Department of Transfusion Medicine, PGIMER, Chandigarh, India

*Background:* Transfusion support is one of the most widely used therapy in hospital practices like any other medical and surgical procedures. Transfusion too has some risks. These may be hemiolytic or non-hemolytic transfusion reactions.

*Aims:* To analyze the spectrum of various transfusion related nonhemolytic reactions and formulate guidelines for better patient care in future by good transfusion practices.

*Materials and Methods:* In a retrospective study with data analysis of period of 3 years (July 2005- July 2008), transfusion related adverse reactions were reported in 90 patients in the department of transfusion medicine, PGIMER, Chandigarh. The relevant data regarding clinical detaikls of patients and laboratory investigations were analyzed.

*Results:* During the study period, 26,400 whole blood units, 87750 packed RBC units and 105291 units of components (platelet concentrate, platelet rich plasma, fresh frozen plasma) were transfused to the patients. Of the 90 adverese events reported, 24 occurred with the whole blood, 62 with PRBC, 03 with FFP and 01 with platelet concentrates.The risk ofacute transfusion reaction with various blood products were: whole blood-1 in 1111, PRBC-1 in 1389, blood components (FFP and PC /PRBC)- 1 in 25,000. The adverse events were categorized as acute hemolytic- 24.4% (n=22), and acute non hemolytic – 75.5% (n=68). The risk ofa cute HTR wasl in 5000 and that of acute non-HTR was 1 in 3333. Of the acute hemolytic events, 22.2% (n=20), were due to improper storage outside the blood bank after issuing and 2 were immune in nature. The acute non hemolytic events comprised of FNHTR -37.7% (n=34), allergic 24.4% (n=22), anaphylactic -11.1% (n=10) and hypwervolemia 2.2% (n=2).

*Conclusion:* Above study revealed that non hemolytic transfusion reactions are more common than acute hemolytic transfusion reactions. Many of these can be minimized with strategy like leucoreduction, appropriate pre-medication and proper inventory management of cellular products. Further, sensitization in training of clinical staff is also necessary for safe bedside transfusion practices.

#### Analysis of transfusion associated adverse events

**S. Basu, R. Kaur, G. Kaur, P. Kaur, S. Sachdev**

Department of Transfusion Medicine, Govt. Medical College and Hospital, Chandigarh, India

*Background:* Unfavorable events occurring during or after transfusion of blood/blood components are not uncommon and constitute adverse transfusion events. In order to ensure safe transfusion practices it is necessary to identify and minimize them. Identification of these events is necessary not only for the patient care but also to rectify existing blood bank protocols and practices.

*Objectives:* To study the incidence of adverse transfusion events and analyze their spectrum in a tertiary care hospital.

*Materials and Methods:* Adverse transfusion events occurring in our hospital were analyzed over 4 years (June 2004 to June 2008). Data was retrieved from transfusion reaction workup files and from patient records.

*Results:* During the study period a total 19,853 whole blood units, 17,038 packed red cell units and 33,726 other component units (platelet concentrates, platelet rich plasma and FFP) were transfused to22412 patients. A total of 283 adverse transfusion events were reported, all were of immediate type. The incidence of adverse transfusion events ranged from 0.81 % to 0.45 % and the commonest was allergic followed by FNHTR. Among the acute hemolytic reactions majority were due to improper storage and faulty administration. There were 2 instances of immune hemolytic transfusion reactions and 3 cases of transfusion associated cardiac overload. Acute reactions due to contaminated products were identified in 4 patients. However no delayed reactions were reported.

*Conclusion:* The decreasing trend incidence of transfusion reactions over the years was probably due to increase in the usage of blood components. Blood Banks need to modify existing management protocols so as to avoid all immune hemolytic transfusion reactions and identify delayed hemolytic transfusion reactions which are presently missed. Majority ofacute hemolytic transfusion reactions occurred due to improper handling and storage. These can be prevented by creating awareness of good beside transfusion practices.

#### Assess the factors hindering the development of protocols for efficient transfusion services by analysing platelet transfusion practices at a tertiary care center

**P. Sinha, D. N. Prasanna**

Department of Transfusion Medicine, Sri Venkateswara Institute of Medical Sciences, Tirupati, India

*Background:* Transfusion services face a problem of setting protocols for correct and efficient transfusion practices. Analyzing the transfusion practices helps us understand the factors that contribute to this problem.

*Material and Methods:* Platelet transfusion practices at our centre were analyzed for a six month period from Jan to Jun 2008 after analyzing patient and blood bank records on files and computer records of hospital. An assessment of requisition forms for platelet components was done to ascertain the completeness and efficacy of communication. An interactive interview with questionnaire with heads of three major user departments was done to analyze the problems faced and possible solutions.

*Results:* 6.6% of all blood component requests received in the blood bank were for platelets. 80% of these requests were incomplete on ≥1 critical entry. 60% of remaining 20% had unauthorized corrected entries. Some entries were never filled.

Transfusion practice comprised of single unit requests (5.4%) and single unit transfusion (16%) and inadequate inventory support in form of inadequate issue (42%). Platelet concentrate utilization is 2.8 units / patient. Average platelet apheresis utilization is 1 / 12 patient (8%); varying from 2 to 17%. Platelet apheresis was not preferred because of cost and need for donors. Some patients, without transfusion, had three different platelet counts reported on different samples drawn on same day.

*Conclusion:* Triggers, Patient assessment, SOP for sampling for platelet counting needs immediate attention. Evaluation of blood requisition form needed, to enforce compliance for critical entries. Need for removing misconceptions about platelet apheresis is felt. Study has given impetus for implementing policies for non group specific platelet transfusion, leucoreduction and pooled platelet products. Apheresis based inventory needs to become cost efficient.

#### Management of blood program

**S. Alexander, V. K. Panicker, R. Krishnamoorthy, R. S. Febe**

Sri Ramachandra University. Chennai, India

*Background:* The heart of all hospitals is the blood bank. No hospital can run without a blood bank and there cannot be a blood bank without blood donors. Human blood is a precious and scarce resource. Donor blood should never go waste. The need of the hour is a proper blood management program to strike a balance between the demand and supply of blood.

*Aim:* To formulate guidelines for balancing the demand and supply of blood through a proper blood management program and ensure the optimal use of blood.

*Materials and Methods:* At the Sri Ramachandra University and Hospital Blood Bank, Porur, Chennai 600 116., which is a 1500 bedded tertiary care hospital there is a voluntary blood donation program to maintain adequate inventory levels. Apart from organizing regular voluntary blood donation camps outside the campus, appeals are made through the public address system within the campus as a result of which students come forward to donate voluntarily. Voluntary non-remunerated repeat blood donation is emphasized and donors are sent reminders about the next date of donation. Regular donors are honored with certificates. Replacement donors also form a part of our donor pool. To ensure that no wastage of human blood occurs, the inventory is managed efficiently. Components are prepared from nearly all donated units. Storage conditions are monitored and maintained. The “first-in-first-out” policy is implemented except in special situations like exchange transfusion. Irradiation is done just before issue of cellular components whenever indicated. Single donor platelets obtained by Apheresis are issued whenever multiple random donor platelet units are requested. Rare donor registries are maintained so that these donors may donate in time of need.

*Results:* Our Blood Bank statistics for the period 2003 August – July 2008 are as follows:

**Table:** 

Voluntary Donors	−	70%
Replacement Donors	−	30%
Component usage	−	98%
Blood utilization rate	−	95%
Blood discard rate	−	5%

*Conclusion:* Voluntary blood donation is the key to safe blood. Voluntary non-remunerated regular repeat blood donation should be emphasized and efforts to retain such donors should be taken. Blood component therapy should be practiced to maximize the utility of single donor unit which will be most beneficial to the patients.

#### Need for mandatory transfusion audit as a tool for quality management in BTS

**A. Jhaveri**

Blood Bank, Indian Red Cross Society, Navsari Distt Branch, Navsari, India

*Background:* An urgent need is always felt for implementation of transfusion audit as a tool to improve clinical transfusion practices for quality management in BTS.

*Aims:* To ascertain whether implementing Transfusion Audits can reduce the unwarranted transfusions and rationalize the use of scarce resource like human blood.

*Material and Methods:* Retrospective analysis of the data of last ten years at Red Cross Blood Bank, Navsari as regards to types of demands received, and quantity of units demanded and actually transfused, units de-reserved and returned with their timings. We also reviewed the relevant literature in this context.

*Results:* Analysis of the retrieved data highlights many important shortcomings on the clinicians’ perspectives, many single unit demands for WHB or components were received. Whole blood is still used where appropriate component can serve the purpose with less possible hazards related to transfusion. Requisition slips are incomplete and/or improper. Blood is more often used without proper indication. PTR are grossly under-reported.

*Conclusion:* Dialogue, consultation and at times confrontation is needed with clinicians in cases of irrational prescriptions. Some legislative involvement is also needed from government agencies to curb misuse of blood. Active participation of end-users in the blood bank activities and functioning is want of time. Hospital transfusion committees need to be set up and regular audits are to be conducted and appropriate prescribing guidelines must be formed and necessary corrections made in mutual dialogue after review of outcome.

#### Whole blood therapy: Criminal waste of most critical human resource

**R. S. Ajmani, R. R. Saraf**

Celestial Biologicals Ltd, Ahmedabad, India

Whole blood therapy is still very rampant in India and it puts severe strain on the transfusion services of the country. All over the world, component therapy is practiced, as it is scientific and rationale way to use the blood. This also has very serious impact on the plasma product program of the country. Therefore scientific and judicious use of this plasma is very important for developing the self sufficiency program for plasma products of the country. As per latest data from CDSCO, India has 2,455 licensed blood banks and about 10-15 % have the license to prepare the blood components. It is estimated that India collect about 75 lakhs unit of blood each year, as against requirement of about 100 lakhs units. Considering the fact that if each unit of collected blood is utilized in most optimum way, it could benefit thousands of patients of haemophilia, infections, organ transplant, shock and burn and many other critical diseases, as their survival is dependent on plasma products. All over the world excess of unutilized human plasma is used for manufacturing of the plasma products. If the same practice is also followed in India, whereby each unit of blood is converted to component and considering 70% of excess plasma is used for preparation of plasma products; we could avail 250 lakhs gm of Albumin, 35 lakhs gm of Immunoglobulins, 750 lakhs I.U. of Factor VIII and many other products. The target of providing the safe plasma products to people of India can only be achieved through strong blood management program of the country, whereby each component of blood is used in the scientific way.

#### Red cell oxidative injury during storage

**R. Chaudhary, R. Katharia, P. Agarwal**

Department of Transfusion Medicine, SGPGIMS, Lucknow, India

*Background:* Storage of red blood cells at 40C is associated with deleterious metabolic and biochemical changes, collectively referred to as “Storage Lesions”. Lipid peroxidation of red cell membrane leading to lysis contributes to these storage lesions. The aim of the present study was to investigate oxidative injury to red cells during storage for 28 days and its correlation with markers of red cell membrane damage.

*Materials and Methods:* Blood samples from 30 red cell units stored at 40C for 28 days were withdrawn aseptically on day 0, day 14 and day 28 of storage. Markers of membrane damage including plasma Hemoglobin, plasma K+, lactate dehydrogenase (LDH) and markers of oxidative injury such as malondialdehyde (MDA) levels, hemoglobin oxidation and osmotic fragility were studied in all samples.

*Results:* A statistically significant *(P=*0.000) increase in the mean values of plasma Hb, plasma K+, LDH and markers of oxidative injury such as MDA and Hb oxidation was observed over the storage period of 28 days. A direct correlation of MDA and Hb oxidation with membrane damage in the form of plasma Hb was observed.

*Conclusion:* Oxidative injury to red cell during storage leads to membrane damage and lysis. Role of antioxidants in prevention of this deleterious effect of storage needs further investigation.

#### The external quality assessment scheme: Five years experience as a participating laboratory

**S. S. Das, R. Chaudhary, S. Ojha, S. Verma, A. Sonker**

Department of Transfusion Medicine, SGPGIMS, Lucknow, India

*Aims and Objective:* Quality assurance in blood banking includes active participation in external quality program. Such program offers valuable benefits to patient care, their safety and overall quality of laboratory practices. In the year 2002, we participated in the External Quality Assessment Scheme (EQAS) under the WHO, Bureau of Laboratory Quality Standards, Thailand.

*Materials and Methods:* In the current study we evaluated our EQAS test result of the past 5 years from 2003 to 2007. Test results of all blood samples such as ABO grouping, D typing, antibody screening, antibody identification and transfusion transmitted infection (TTI) testing were analyzed and documented.

*Results:* Discordant results in one or more instances were observed with antibody identification, weak D testing and tests for anti-HIV1/2 and HBsAg. Twice we failed to detect ’anti-Mia’ antibody in the issued sample and that could be attributed to the absence of corresponding antigen in the used cell panel. HBsAg was missed due to its critically low titer in the serum and a comparatively low sensitivity of our ELISA test kit.

*Conclusion:* All these failures in the last 5 years helped us significantly to improve our transfusion service in terms of performance evaluation, patient care and safety issues and overall quality of laboratory practices. We therefore recommend all laboratories and hospitals to participate in the EQAS program which will definitely help them to improve from what they learn.

#### A study of T cell CD4, CD8, CD28 protein expression in blood iron overloading condition

**Alireza Andalib, Pourazar Abbasali, Rezaei Abbas, Moayedi Behjatsadat, Bagherpour Bahram**

Immunology Department -Isfahan Medical School, Isfahan, Iran, India

*Background:* Pathological situation may affect cell protein expression. Transfusional iron overload is a most common metal-related toxicity condition. Aim: to evaluate the blood iron overloading condition on T cell receptor, CD4, CD8, CD28 protein expression, the present study was designed

*Methods and materials:* PBMCs were taken from thalassemia patients and healthy control group. The lymphocytes were isolated using Ficoll-Hypaque gradient technique (lymphoprep; Norway). Mouse monoclonal anti-CD28 antibody conjugated with phycoerythrin (serotec, UK), and anti-CD4, -CD8 antibody conjugated with FITC were used. Staining lymphocytes were analyzed by FACScalibour flow cytometer (Becton-Dickinson, USA), the data obtained were analyzed using a cell Quest software installed in the flow system, and then the obtained data was analyzed by SPSS. A tissue culture model was designed for evaluating an in vitro iron overloading condition to compare with *in vivo* situation.

*Results:* the percentage of TCD4+ cell in control group was determined %48.3 ± 5.3 and in patient group was %44.2± 4.9. Moreover, CD8+ lymphocytes in thalassemia patient group were %21.8 ± 2.41 and in healthy group was counted % 25.04±3.25. The frequency of CD28 expression on T cell CD4+ in patient group was %38.7±2.98 and in control group was determined %43.7±4.39. In addition, on T CD8+ cells the frequency of CD28+ expression was %21.8±2.41 in thalassemia group, but it was %12.03 ± 3.6 in the control group. The ratio of CD4+/CD8+ in thalassemia and control group were 1.52±0.21 and 2.1±0.24 respectively. *Conclusion:* It seems that blood with iron overload condition could effect the lymphocyte protein expression as well as decrease the cell signaling protein, particularly for CD8+ lymphocytes.

